# How to Estimate Epidemic Risk from Incomplete Contact Diaries Data?

**DOI:** 10.1371/journal.pcbi.1005002

**Published:** 2016-06-24

**Authors:** Rossana Mastrandrea, Alain Barrat

**Affiliations:** 1 Aix Marseille Univ, Univ Toulon, CNRS, CPT, Marseille, France; 2 IMT Institute of Advanced Studies, Lucca, Lucca, Italy; 3 Data Science Laboratory, ISI Foundation, Torino, Italy; Ecole Polytechnique Federale de Lausanne, SWITZERLAND

## Abstract

Social interactions shape the patterns of spreading processes in a population. Techniques such as diaries or proximity sensors allow to collect data about encounters and to build networks of contacts between individuals. The contact networks obtained from these different techniques are however quantitatively different. Here, we first show how these discrepancies affect the prediction of the epidemic risk when these data are fed to numerical models of epidemic spread: low participation rate, under-reporting of contacts and overestimation of contact durations in contact diaries with respect to sensor data determine indeed important differences in the outcomes of the corresponding simulations with for instance an enhanced sensitivity to initial conditions. Most importantly, we investigate if and how information gathered from contact diaries can be used in such simulations in order to yield an accurate description of the epidemic risk, assuming that data from sensors represent the ground truth. The contact networks built from contact sensors and diaries present indeed several structural similarities: this suggests the possibility to construct, using only the contact diary network information, a surrogate contact network such that simulations using this surrogate network give the same estimation of the epidemic risk as simulations using the contact sensor network. We present and compare several methods to build such surrogate data, and show that it is indeed possible to obtain a good agreement between the outcomes of simulations using surrogate and sensor data, as long as the contact diary information is complemented by publicly available data describing the heterogeneity of the durations of human contacts.

## Author Summary

Schools, offices, hospitals play an important role in the spreading of epidemics. Information about interactions between individuals in such contexts can help understand the patterns of transmission and design ad hoc immunization strategies. Data about contacts can be collected through various techniques such as diaries or proximity sensors. Here, we first ask if the corresponding datasets give similar predictions of the epidemic risk when they are used to build a network of contacts among individuals. Not surprisingly, the answer is negative: indeed, if we consider data from sensors as the ground truth, diaries are affected by low participation rate, underreporting and overestimation of durations. Is it however possible, despite these biases, to use data from contact diaries to obtain sensible epidemic risk predictions? We show here that, thanks to the structural similarities between the two networks, it is possible to use the contact diaries to build surrogate versions of the contact network obtained from the sensor data, such that both yield the same epidemic risk estimation. We show that the construction of such surrogate networks can be performed using solely the information contained in the contact diaries, complemented by publicly available data on the heterogeneity of cumulative contact durations between individuals.

## Introduction

Knowledge of the structure of human interactions is crucial for the study of infectious diseases spread and the design and evaluation of adequate containment strategies. The structure of contact networks, [1], the presence of communities [2], bridges or “linkers” between communities [3–5], “super-spreaders” [6–8], the heterogeneity of contact durations [9], are all important characteristics that determine potential transmission patterns. The study of human contacts is particularly relevant in contexts such as schools, working places, hospitals where individuals might spend several hours in close proximity [5, 10–22].

Interactions and contacts between individuals are conveniently seen within the framework of networks in which nodes represent individuals and (weighted) links correspond to the occurrence of contacts (the weight giving the duration of the contacts). Measuring directly such networks represents an important challenge [20]. Many studies have relied on contact diaries or surveys [10, 12, 23–32], while technological advances have led to a strong increase in the use of wearable sensors in the recent years [9, 13–15, 17–20, 33–35]. Quantitative comparisons between datasets obtained from sensors and self-reported diaries, in terms of the numbers and durations of contacts between individuals and of the contact network statistics, are however scarce, mainly because very few studies have combined these two data collection means [21, 36]. These investigations have shown that diaries suffer from small participation rates, under-reporting of contacts, and over-estimation of the contact durations. Under-reporting is particularly strong for short contacts, while long ones are better reported, and some studies have put forward methods to estimate its magnitude and to correct for it [29, 37]. Interestingly, and despite the much lower number of nodes and links in contact networks inferred from contact diaries, the overall structure of these networks is very similar to the one obtained from wearable sensors. Moreover, the links with largest weights (as measured by sensors), which might play a major role in propagation processes, are reported with high probability in the contact diaries.

In this paper, we go beyond the comparison of the contact networks obtained by these methodologies and explore the impact of their differences on the evaluation of the epidemic risk when such datasets are used in numerical simulations of infectious disease propagation. Our goal is to understand to what extent and how the information gathered from contact diaries can be used in such simulations in order to yield an accurate description of the epidemic risk, despite the biases mentioned above. We first compare the outcomes of spreading simulations performed using data coming from wearable sensors and from contact diaries that describe the contacts between students in the same context (a high school) and on the same day. Although the two networks are supposed to describe the same reality, we observe important differences in the simulations, due to the low participation rate in the diaries and to a stronger community structure in the contact diaries network than in the contact sensors network. We then design and evaluate a set of methods to use the information contained in the contact diaries to build surrogate versions of the contacts that yield, when used in the simulations, a better estimation of the real epidemic risk as quantified by the distribution of epidemic sizes (considering as ground truth the dataset from the sensors). We show that good results are obtained when the contact diary information is complemented by known stylized facts characterizing human interactions, in particular the heterogeneity of contact durations.

## Results

### Data description

We use two datasets collected in a French high-school in 2013 and made publicly available in [21]. The data describe face-to-face contacts between students of 9 classes as collected by (i) the SocioPatterns infrastructure [38] based on wearable sensors, during one week and (ii) self-reported contact diaries filled on a specific day of the same week (Dec. 5^th^, 2013). In the diaries, contact was explicitly defined as close (less than 2 m) face-to-face proximity, in order to match as much as possible this definition to the contacts detected by sensors. Using these data, we build two distinct contact networks for the day in which the diaries were collected: the Contact Sensors Network (CSN) and the Contact Diaries Network (CDN). In each network, nodes represent students and a link is drawn between a pair of nodes (*i*, *j*) if at least one contact between students *i* and *j* is present in the corresponding dataset during the considered day. We present and compare in the Supporting Information the main networks’ characteristics. Note that, in the diaries, some participants reported contacts with non-participants. One could a priori use this information and build a contact network including both participants and non-participants. However, since by definition the contacts of non-participants are unknown, this would introduce a potentially strong and most importantly completely uncontrolled bias in the measures of the network’s structural properties such as, e.g., its clustering or the node degrees.

A weight can moreover be assigned to each link (*i*, *j*): for the CSN, the weight *w*_*ij*_ is given by the cumulative duration of the contacts registered by the sensors on that day between *i* and *j*; for the CDN we can use the duration reported by the students in the diaries, building the network CDN_D_, or use for each link a duration taken at random from the list of durations registered by the sensors, obtaining the network CDN_S_ (see Methods for details). The rationale behind building CDN_S_ comes from the results of [21, 36] that show that durations reported by students tend to be strongly overestimated. Since, on average, contacts reported in the diaries as long tend also to be long according to the sensor data, we will also consider a different assignment of links to the CDN, in which we still take durations at random from the list of durations registered by the sensors, but assign the longer durations to the links of CDN with longer reported durations: we denote the resulting network by CDN_S’_.

The contact sensor network counts 295 nodes (participation rate 77.8%) and 2162 links, while the contact diaries network has 120 nodes (participation rate 31.6%) and 348 links. Incomplete participation, even in the case of the sensor data, leads to biases in the simulations using the CSN with respect to what would be obtained if the whole population had participated, due to the fact that contacts with and among non-participants are not detected. This point has been discussed in [40], together with methods to build surrogate data and obtain estimate of the epidemic risk in the case of such population sampling. In order not to confuse the issues of population sampling and comparison between diaries and sensors, we consider here as ground truth the CSN, collected by wearable sensors for which the definition of contact does not depend on a possible interpretation of the diary question by the students nor on the fact that they might not recall contact events.

### Numerical simulations of epidemic spread

In the following, we perform simulations of the spread of infectious diseases in the considered population, using as substrate for propagation events the contact networks described above. It is important to note here that we consider propagation processes on static networks. Indeed, the CDN does not contain information on the timing of the contacts, so that it is natural to compare the outcome of simulations performed on such a static network with simulations performed on a static version of the CSN. Moreover, when modeling the propagation of infectious diseases with realistic timescales of several days, it has been shown in [39] that a daily weighted contact network contains enough information to obtain a good estimate of the process outcome. When dealing with faster processes, the temporal evolution of the network would become relevant; in that case, it would be possible to use the techniques put forward in [40] to build realistic surrogate timelines of contacts on weighted networks, using the robustness of the distributions of the durations of single contact events and of the intervals between successive contacts measured in different contexts. Note also that, even if the networks do not take into account the timing of the contact events, they still include information on the aggregate contact durations through the weights, which are known to play a crucial role in the outcome of spreading processes [39, 41–43].

For simplicity, we consider the paradigmatic Susceptible-Infected-Recovered (SIR) model of epidemic propagation. In this model, each Susceptible node *i* can be infected by an Infected neighbour *j* with probability β*w_ij_*dt for each small time step *dt*. Infected people recover with rate *μ* and enter in the Recovered category. Recovered individuals cannot be infected again. The process starts with a single Infected individual chosen at random (the seed) and ends when there are no more Infected nodes. The epidemic risk in the population, which depends on the interplay of the ratio *β*/*μ* and the network’s structure and weights, is measured by the distribution of the final size of epidemics (i.e., of the fraction of individuals in the Recovered category at the end of the process), obtained by repeating the simulations with randomly chosen seeds. Note that, since we consider static networks, only the ratio *β*/*μ* is relevant, and multiplying both by a certain factor only changes the timescale on which the epidemic unfolds. The shape of the distribution of epidemic sizes depends on the features of the underlying network structure in terms of possible patterns of contagion. The comparison of these distributions gives hints about similarities and discrepancies of various datasets for the evaluation of the epidemic risk.

**Fig 1:**
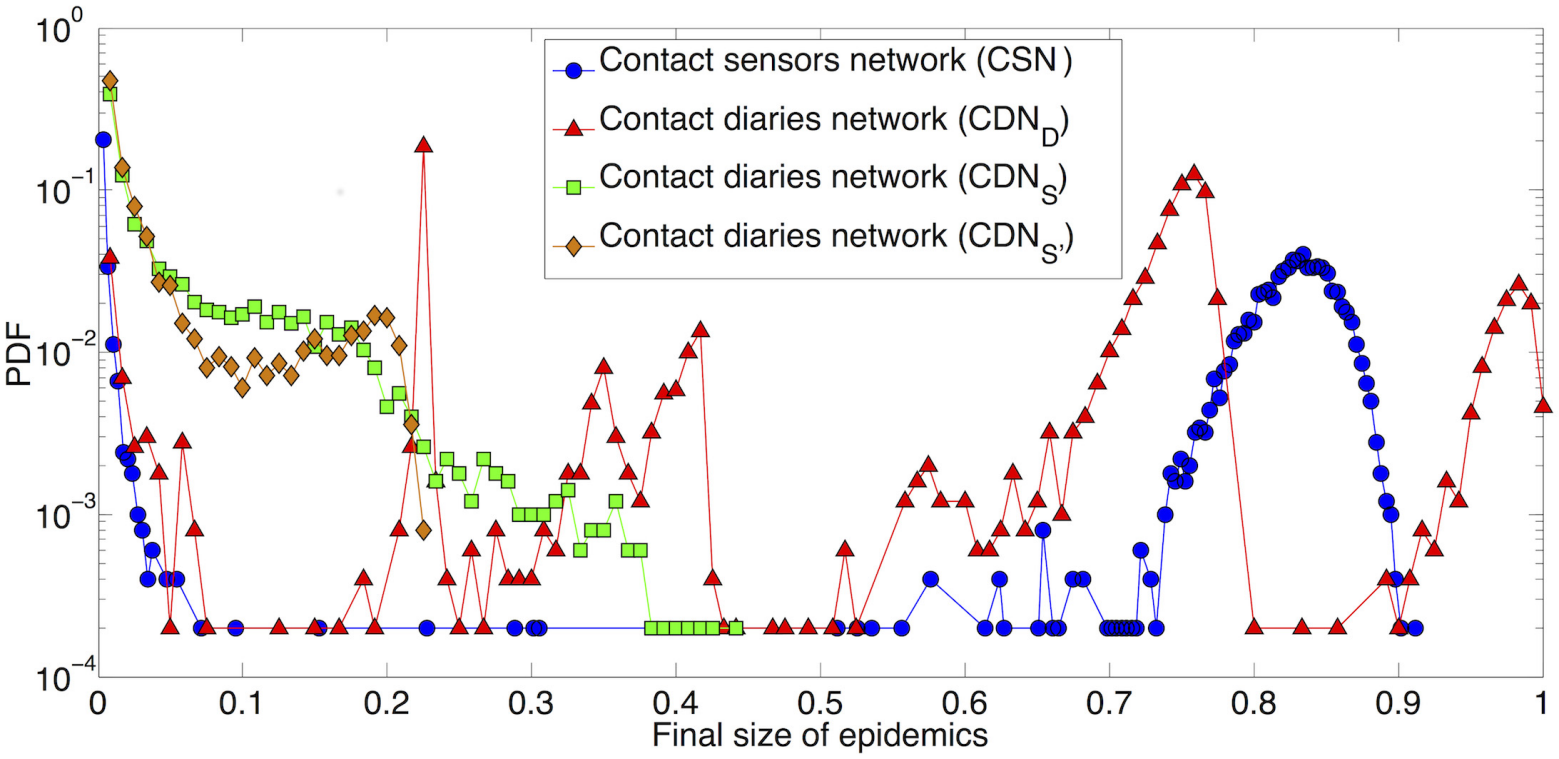
Distribution of final size of epidemics. 1000 SIR simulations performed on the original contact sensors network (CSN) and the original contact diaries network with durations respectively reported by students (CDN_D_) and registered by sensors (CDN_S_ and CDN_S’_). Each process starts with one random infected seed. *β*/*μ* = 30.

We first compare in Fig 1 the outcome of simulations of the SIR model performed on the CSN and on the two versions of the CDN described above (CDN_D_ with weights reported by students and CDN_S_ with weights registered by sensors assigned randomly to the links), for one specific value of *β*/*μ* = 30. The three distributions of epidemic sizes are very different from each other. The outcome of simulations performed using CSN is quite standard, with a fraction of small outbreaks that reach only a small fraction of the population and another peak corresponding to large outbreaks. As shown in the Supporting Information, the outcome does not depend on the class of the initial seed. The shape of the distribution obtained when using the CDN_D_ is more peculiar, with a series of peaks, including one at very large epidemic sizes. Such structure is typical of spreading processes on networks with a strong community structure [4], which corresponds to the results of [21]: (i) due to the low participation rate and the under-reporting, the community structure of the CDN is stronger than the one of the CSN, with few links between classes; depending on the seed, the simulated disease can thus remain confined in one class or in a group of few classes, leading to the peaks at intermediate values of the epidemic size; we moreover show in the SI that the outcome depends on the class of the initial seed for the CDN but not for the CSN; (ii) on the other hand, as contact durations are overestimated, the propagation probability on each link is also overestimated and, if the disease manages to spread between classes, almost all individuals are affected, leading to the peak at large epidemic sizes. The CDN_S_ case shows a different result: no more than half of the whole population is affected by the spread. As the weights have in this case the same statistics as the CSN, this is simply due to the low participation rate [40] and the much smaller average degree in the CDN with respect to the CSN. We also note that, since the weights are assigned randomly to the links between students, the structure of the contact matrix giving the average durations of contacts between students of different classes can strongly differ between the CDN_S_ and both the CSN and the CDN_D_, leading to different patterns of propagation between classes (see Supporting Information). We finally note that the simulations on the CDN_S’_, which keeps the distribution of the weights from CSN and in which larger weights are assigned to links with longer reported durations, yield even smaller outbreaks. This is probably due to the fact that the large weights reported in the diaries tend to be within classes, so that the links bridging classes and favoring the spread tend to have smaller weights in the CDN_S’_ than in the CDN_S_. We also show in the SI the temporal evolution of the density of infectious individuals for the various cases considered here.

#### Matching networks

In order to discard the differences due simply to the population sampling, and to focus on the impact of under-reporting (i.e., of unreported links) and overestimation of durations on the estimation of the epidemic risk, we consider “matched” versions of the networks, in which we keep only the nodes present in both CSN and CDN. We obtain the *matched* networks: CSN^*m*^, ${\text{CDN}}_{\text{D}}^{m}$ and ${\text{CDN}}_{\text{S}}^{m}$. Among the 120 students who filled in the diaries, 11 in fact did not wear sensors on the day of interest: we obtain thus 109 nodes, distributed in 7 of the 9 classes of the CSN. We moreover discard one of the classes in which only one student filled in the diary. We end up with matched networks of 108 nodes in 6 classes.

**Fig 2:**
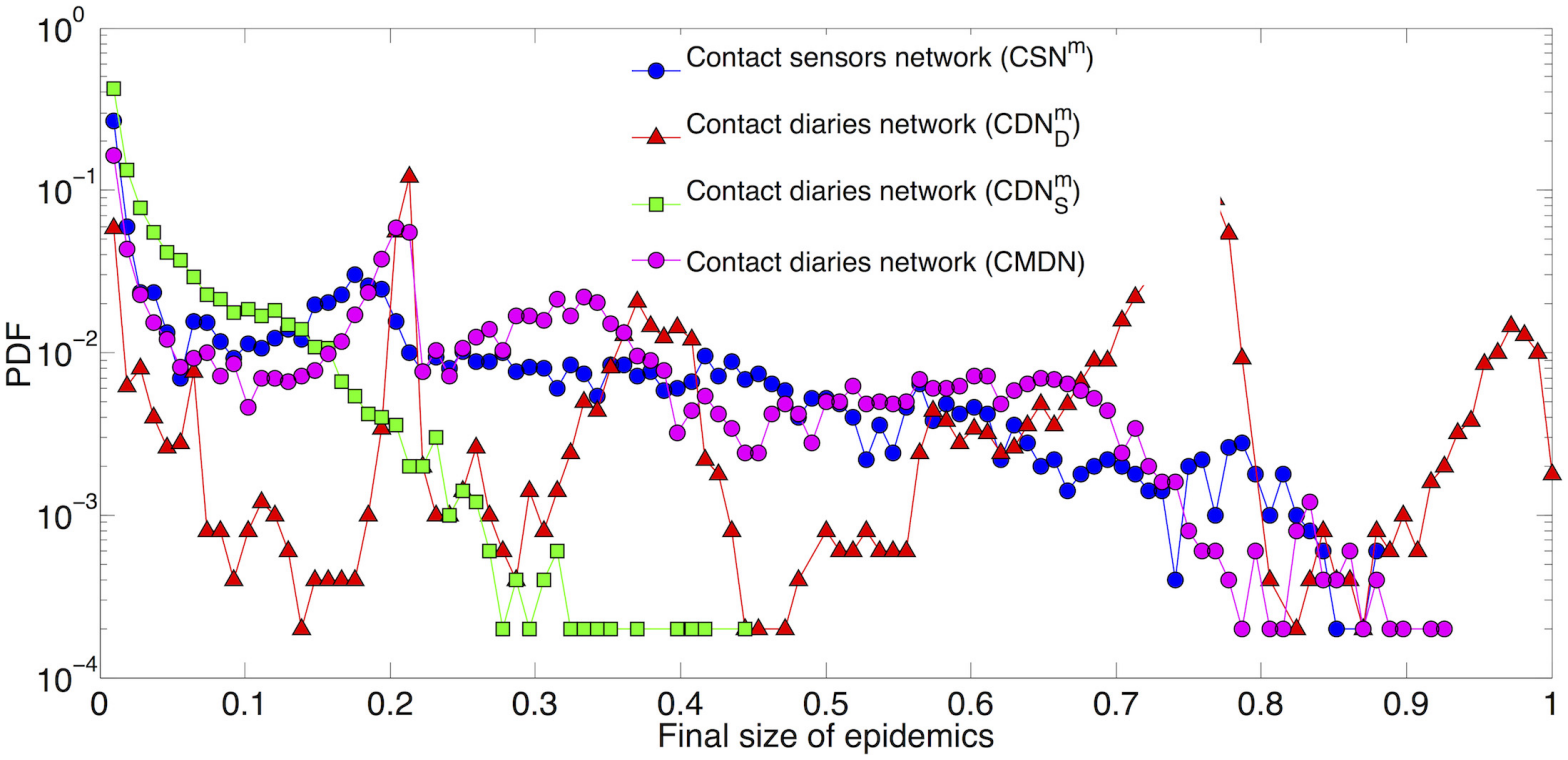
Distribution of final size of epidemics. 1000 SIR simulations performed on the matched contact sensors network (CSN^*m*^), the matched contact diaries network with durations respectively reported by students (${\text{CDN}}_{\text{D}}^{m}$) and registered by sensors (${\text{CDN}}_{\text{S}}^{m}$) and the contact diaries network with weights obtained by a negative binomial fit of contact durations reported by students between and within classes (CMDN). Each process starts with one random infected seed. *β*/*μ* = 30.

Fig 2 displays the outcome of SIR simulations performed on the three matched networks. Comparison with Fig 1 shows that the outcomes for ${\text{CDN}}_{\text{D}}^{m}$ and ${\text{CDN}}_{\text{S}}^{m}$ are similar to the cases of CDN_D_ and CDN_S_, which is expected as these networks do not differ strongly (only 12 nodes and 62 links have been removed in the matching procedure). On the other hand, the epidemic risk is strongly underestimated in the CSN^*m*^ with respect to the CSN: this is due to the strong reduction in the number of nodes and links [40] and hence in the number of potential transmission routes between students and classes. However, the distribution does not exhibit peaks as for the ${\text{CDN}}_{\text{D}}^{m}$ case: the community structure remains indeed weaker in the CSN^*m*^ with respect to the CDN^*m*^, with higher densities of links between different classes. We also note that the underestimation obtained by using CSN^*m*^ is less strong than in the case of a random removal of the same number of nodes [40] (not shown). This is due to the fact that the students who filled in the diaries tend to be more connected than the others in the CSN: as a result, the CSN^*m*^ has 970 links while a random removal of the same number of nodes from the CSN leads on average to a network with ≈560 links. Finally, although both CSN^*m*^ and ${\text{CDN}}_{\text{S}}^{m}$ have the same distributions of weights and lead both to strong underestimations of the epidemic risk, the resulting distributions do not coincide, in particular because the ${\text{CDN}}_{\text{S}}^{m}$ has a much smaller number of links.

We also show in Fig 2 the outcome of simulations performed using another representation of the contact diaries network, namely the Contact Matrix Distribution Network (CMDN) introduced in [44] and built as follows: as explained in Methods, we perform a fit of the distributions of contact durations reported by students by a negative binomial functional form, distinguishing between contacts between students of the same class or of different classes. We then use these fitted distributions to randomly assign weights to each pair of students. Note that these weights can be equal to 0, in which case no link is drawn between the students. This procedure yields a network with global link density close to the CDN^*m*^ and such that the contact matrices of link densities and of average contact durations between classes are also similar to the ones obtained from the CDN^*m*^ (see Supporting Information). The overall result is a distribution of epidemic sizes more similar to the case of the CSN^*m*^ (results for other values of *β*/*μ* are shown in the Supporting Information).

#### Building surrogate networks to estimate the epidemic risk

We now address the issue of how the data coming from contact diaries could be used to provide an accurate estimation of the epidemic risk, despite the discrepancies obtained when these data are used directly in simulations. To this aim, we propose several procedures to build surrogate contact diaries networks that overcome the issues of low participation rate and overestimation of contact durations, which bear a strong impact on the simulation outcome, as shown above. In the same spirit as [40], we start from the available dataset and extend it by adding the missing nodes to the contact network, as well as surrogate links. We build these surrogate networks using only information known in the CDN^*m*^. We note that we do not try to infer the true missing links but to build a “plausible” version of these links, such that the simulations of epidemic spread on the resulting network yield an accurate estimation of the epidemic risk.

**Fig 3:**
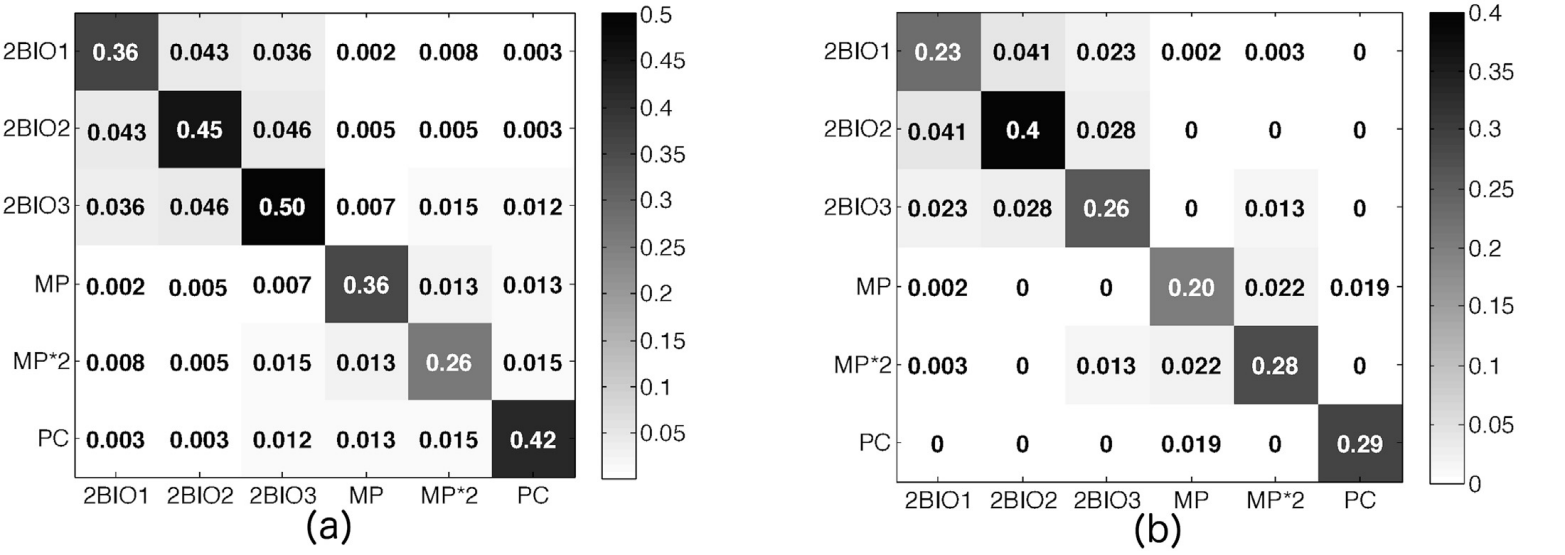
Contact matrices of edge densities. The entry at row *X* and column *Y* of the matrix is given by the total number of links between students in class *X* and students in class *Y*, normalized by the maximum number of observable links (*n*_*X*_ *n*_*Y*_ or *n*_*X*_(*n*_*X*_ − 1)/2 if *X* = *Y*, with *n*_*X*_ the cardinality of the class *X*) giving the edge link densities for (a) the contact sensors network and (b) the matched contact diaries network, both limited to the 6 classes considered. The cosine similarity between the two matrices is equal to 97%.

The rationale behind the procedures we propose comes from (i) the observed similarity between the overall structure of the contact networks measured by sensors and by diaries, as quantified by the contact matrices of the densities of links between classes [21] shown in Fig 3, and (ii) the results of [44] that show how such contact matrices, together with information on the heterogeneity of contact durations, play a crucial role in determining propagation patterns in a structured population. Note that, as 3 of the 9 classes of the CSN are not present in the CDN, we consider here a version of the CSN limited to the remaining 6 classes (the resulting CSN has 204 nodes and 1600 links).

We propose a three-steps procedure to build surrogate contact networks for the 6 classes considered, starting from the CDN^*m*^, which contains only a fraction of the nodes of these classes (see details in Methods): (i) we first add the missing nodes in each class; (ii) we randomly add links in each class and between classes in order to maintain the contact matrix of edge densities fixed to its measured value in the CDN^*m*^, shown in Fig 3(b); (iii) we associate weights to the links of the resulting surrogate network CDN^*s*^.

Both steps (ii) and (iii) can be performed in different ways. With respect to step (ii), we notice that the empirical contact matrix (Fig 3(b)) contains some elements equal to zero, corresponding to a total absence of links between classes. This corresponds to an unrealistically strong community structure and is due to the low sampling rate and to the under-reporting of contacts. We thus consider two cases: (a) we strictly keep the contact matrix with its zero elements; (b) we replace the zeros with random values drawn from a uniform distribution between the minimum and maximum values of the non-zero off-diagonal elements of the matrix (see Methods). In what follows, we will refer to these cases respectively with the subscripts *z* (‘zero’) and *nz* (‘no zero’), obtaining ${\text{CDN}}_{\text{z}}^{s}$ and ${\text{CDN}}_{\text{nz}}^{s}$.

**Fig 4:**
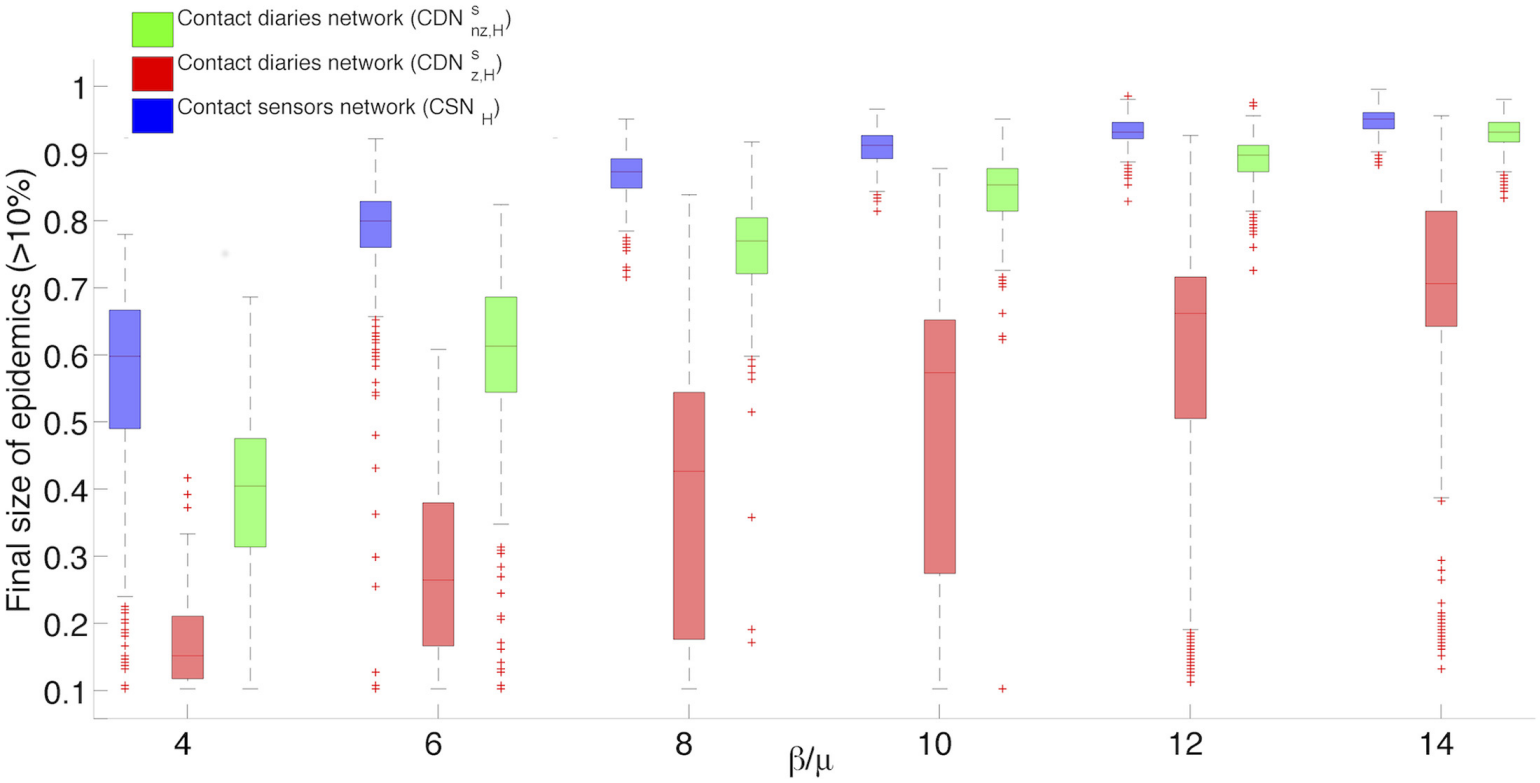
Box-plots of epidemic size distributions. Comparison of the distributions of epidemic sizes for epidemics reaching more than 10% of the population, resulting from SIR simulations performed on the contact sensors network with homogeneous weights (CSN_H_) and two surrogate contact diaries networks with homogeneous durations (${\text{CDN}}_{\text{z,H}}^{s}$, ${\text{CDN}}_{\text{nz,H}}^{s}$). For each boxplot, the central mark stands for the median, its edges represent the 25^th^ and 75^th^ percentiles. The whiskers extend to the most extreme data points not considered outliers, while outliers are plotted individually. Points are drawn as outliers if they are larger than *a*+*h*(*b* − *a*) or smaller than *a* − *h*(*b* − *a*), where *a* and *b* are the 25th and 75th percentiles, respectively and *h* is the maximum whisker length set by default to *h* = 1.5. (1000 simulations for each value of the ratio *β*/*μ* ∈ {4, 6, 8, 10, 12, 14}).

We first focus on the structure obtained through this procedure. We show in the SI some statistical characteristics of the surrogate networks, compared to the empirical CDN and CSN: in particular, the structural properties of ${\text{CDN}}_{\text{nz}}^{s}$ are much closer to the CSN than the empirical CDN. Moreover, we start by simply assigning homogeneous weights in step (iii) and compare the outcome of simulations of the SIR model with simulations performed on a version of the CSN with as well homogeneous weights, denoted CSN_H_. This amounts to the assumption that each student spends the same amount of time with all his/her contacts, a minimal assumption corresponding to an absence of information about contact durations. We report in Fig 4 boxplots for the distributions of epidemic sizes larger than 10%, obtained from SIR simulations at various values of *β*/*μ* on the resulting homogeneous networks ${\text{CDN}}_{\text{z,H}}^{s}$ (homogeneous weights and contact matrix zeros kept) and ${\text{CDN}}_{\text{nz,H}}^{s}$ (homogeneous weights and contact matrix zeros replaced). We also report in the Supporting Information the fraction of epidemics reaching more than 10% of the population, as a function of *β*/*μ*.

Fig 4 and Fig S7 in S1 File clearly show that, despite the high similarity between the contact matrices of the surrogate network and of the CSN, an important underestimation of the epidemic size is obtained. Replacing the zeros in the contact matrix gives better results but still yields a clear underestimation of the risk with respect to the CSN reference.

Let us now turn to the more realistic hypothesis of heterogenous cumulative durations of contacts among students. It is indeed known that these durations are very heterogeneous: most are short, but durations orders of magnitude longer than the average are not uncommon [9]. Within the usual hypothesis of a transmission probability proportional to the contact duration, this implies that different contacts can correspond to very different transmission probabilities, and hence that they should not be treated as equivalent. The importance of taking the diversity of contact durations has indeed been assessed for instance in [39, 41–43]. In this case, step (iii) of the surrogate network building procedure, which regards the assignment of weights to links, needs to be precised. We consider here two possibilities: we use either the list of weights (daily cumulative durations) reported in the diaries, or the list of weights registered by the sensors. In both cases, weights are randomly drawn from the empirical list and assigned at random to the links in the surrogate network (see Methods). Taking into account the two possibilities of keeping or replacing the zeros in the contact matrix of link densities, we end up with four surrogate contact networks: ${\text{CDN}}_{\text{z,D}}^{s}$ and ${\text{CDN}}_{\text{nz,D}}^{s}$, both with weights randomly drawn from the list of durations reported by students (note that we do not find different results between keeping fixed the original weighted structure of the CDN and assigning random weights also to the corresponding links); ${\text{CDN}}_{\text{z,S}}^{s}$ and ${\text{CDN}}_{\text{nz,S}}^{s}$, both with weights randomly picked from the cumulative durations registered by sensors.

**Fig 5:**
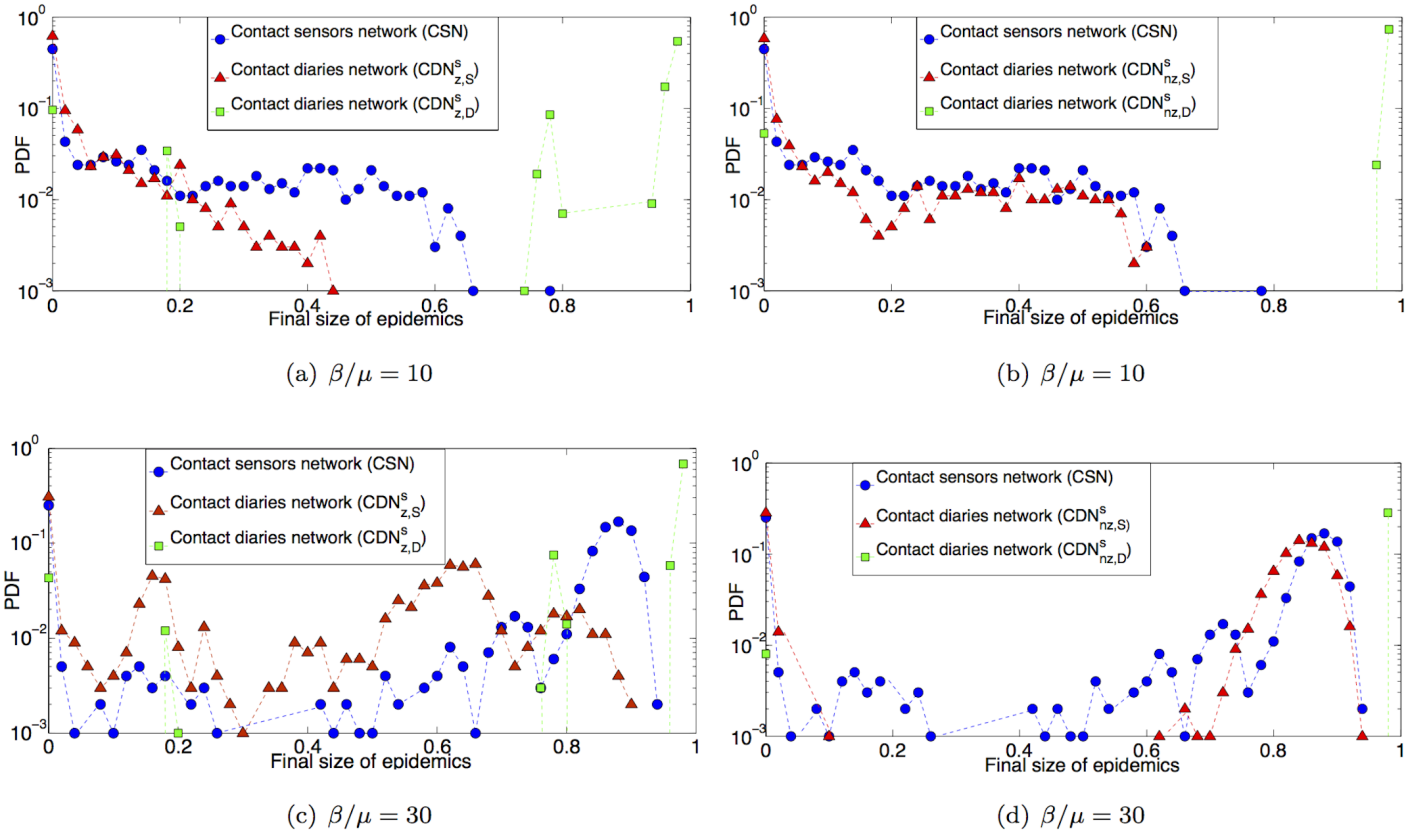
Distribution of final size of epidemics. 1000 SIR simulations performed on the contact sensors network (CSN) and the four surrogate contact diaries network with and without zeros in the contact matrix of link densities, and with durations extracted at random from the lists of values respectively reported by students and registered by sensors: (a), (c) ${\text{CDN}}_{\text{z,D}}^{s}$ and ${\text{CDN}}_{\text{z,S}}^{s}$; (b), (d) ${\text{CDN}}_{\text{nz,D}}^{s}$ and ${\text{CDN}}_{\text{nz,S}}^{s}$. Each process starts with one random infected seed. *β*/*μ* ∈ {10, 30}.

Fig 5 presents the outcome of SIR simulations on these surrogate networks, compared to the distributions of epidemic sizes obtained with the CSN, for two values of *β*/*μ*. First, the overestimation of the contact durations in the diaries, combined with the replacement of zeros in the contact matrix, leads to a very strong overestimation of the epidemic risk when ${\text{CDN}}_{\text{nz,D}}^{s}$ is used. The ${\text{CDN}}_{\text{z,D}}^{s}$ in turn yields a peculiar shape of the distribution with intermediate peaks, due to its unrealistically strong community structure, in a way similar to the CDN_D_ case. Distributions obtained with ${\text{CDN}}_{\text{z,S}}^{s}$ are also impacted by this structure and lead to an underestimation of the risk together with the intermediate peaks due to the strong community structure. Finally, simulations performed using the ${\text{CDN}}_{\text{nz,S}}^{s}$ give a much better prediction of the epidemic risk associated to the CSN (Fig 5(b) and 5(d)).

Some differences between the outcomes of simulations using the contact sensor network and the ${\text{CDN}}_{\text{nz,S}}^{s}$ are nonetheless observed at large *β*/*μ*, in particular for intermediate epidemic sizes (epidemics involving between 20% and 70% of the population): a non-negligible contribution to the epidemic size distribution is observed for the CSN but not for the ${\text{CDN}}_{\text{nz,S}}^{s}$. We show in the Supporting Information that the distribution of sizes obtained on a version of the CSN in which weights are randomly reshuffled looses this contribution of intermediate size epidemics. This shows that the observed discrepancies result from the random assignment of weights to links in the ${\text{CDN}}_{\text{nz,S}}^{s}$, which does not preserve correlations between structure and weights present in the CSN.

**Fig 6:**
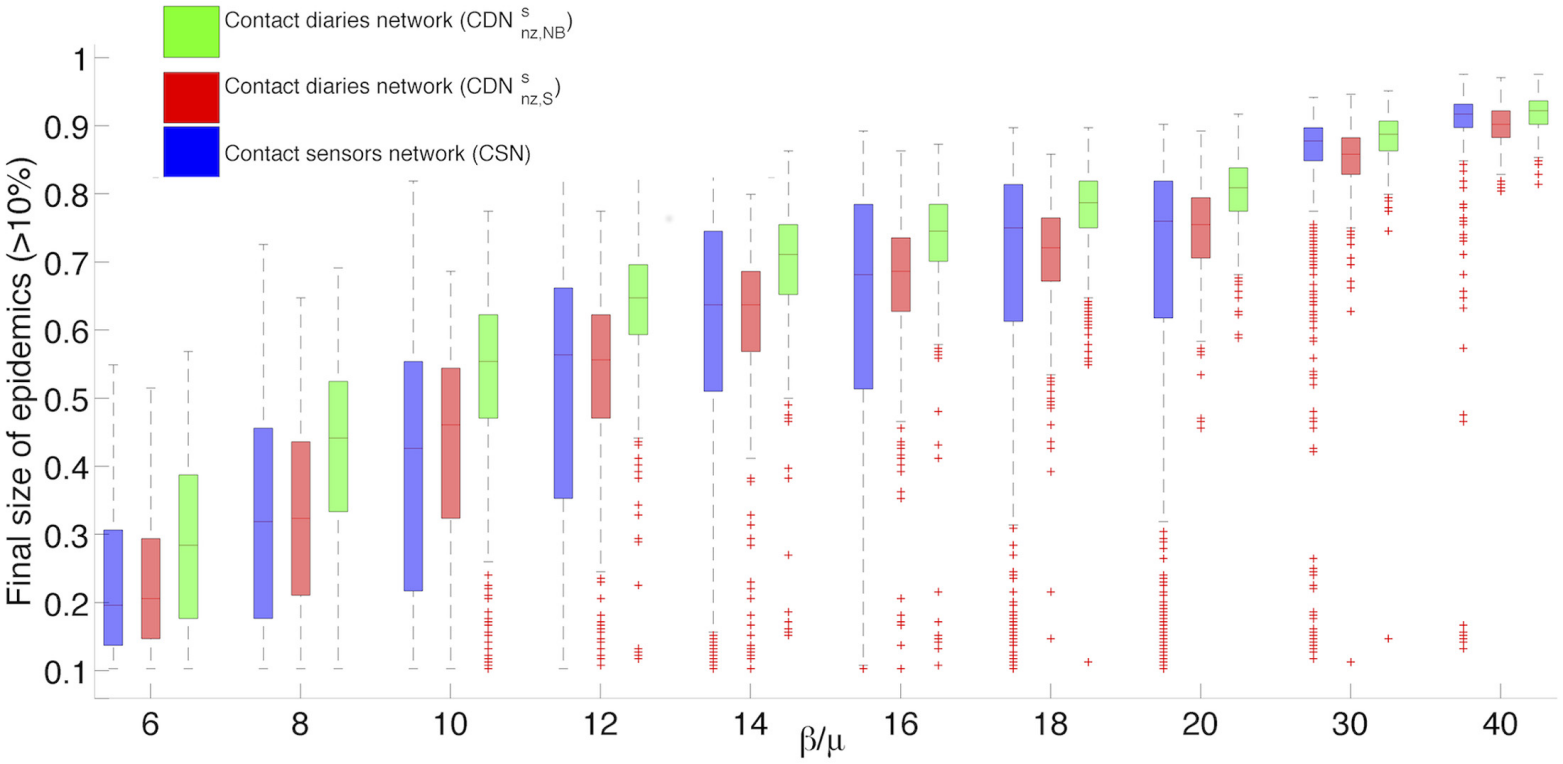
Box-plot of the distribution of epidemic sizes larger than 10%. Comparison of the distribution of epidemic sizes for SIR simulations performed on the contact sensors network (CSN), the surrogate contact network without zeros in the contact matrix of link densities and with weights randomly drawn from the distribution of contact durations registered by sensors (${\text{CDN}}_{\text{nz,S}}^{s}$), and the same surrogate contact network but with weights randomly drawn from a negative binomial fit of the distribution of contact durations registered by sensors in several similar environments (${\text{CDN}}_{\text{nz,NB}}^{s})$. For each box, the central mark stands for the median, its edges represent the 25^th^ and 75^th^ percentiles. The whiskers extend to the most extreme data points not considered outliers, while outliers are plotted individually. Points are drawn as outliers if they are larger than *a* + *h*(*b* − *a*) or smaller than *a* − *h*(*b* − *a*), where *a* and *b* are the 25th and 75th percentiles, respectively and *h* is the maximum whisker length set by default to *h* = 1.5. (1000 simulations for each value of the ratio *β*/*μ*. *β*/*μ* ∈ {6, 8, 10, 12, 14, 16, 18, 20, 30, 40}).

Fig 6 shows the robustness of our results concerning the comparison of outcomes of simulations on the various networks when *β*/*μ* is varied, by presenting the boxplots of the distribution of epidemic sizes for epidemics that involve more than 10% of the population. The fraction of such epidemics as a function of *β*/*μ* is shown in the Supporting Information. Overall, a good agreement is observed for all values of *β*/*μ*, with however a systematic small underestimation of the largest epidemic sizes as well as an underestimation of intermediate sizes, especially at large *β*/*μ* (see Supporting Information), and a narrower peak at large sizes when the ${\text{CDN}}_{\text{nz,S}}^{s}$ is used.

In order to build ${\text{CDN}}_{\text{nz,S}}^{s}$, we have used in step (iii) the distribution of aggregate contact durations measured by the sensors. We however need to consider the possibility that only diaries have been collected in a given setting, so that such a distribution is not available. To this aim, we take advantage of the robustness of such distributions, as discussed for instance in [9]. We investigate this issue in some more details here, to understand if distributions of contact durations are similar enough in different contexts: our aim is to use publicly available data on contact duration distributions in a context-independent way in the step (iii) of our procedure. We consider five publicly available datasets, corresponding to contacts measured by wearable sensors in: a French and an American primary school [35, 45], an office building [5] a hospital [22] and a conference [39]. All these data have been collected by the SocioPatterns collaboration [38], except for the case of the American primary school, in which a different infrastructure was used [35].

**Fig 7:**
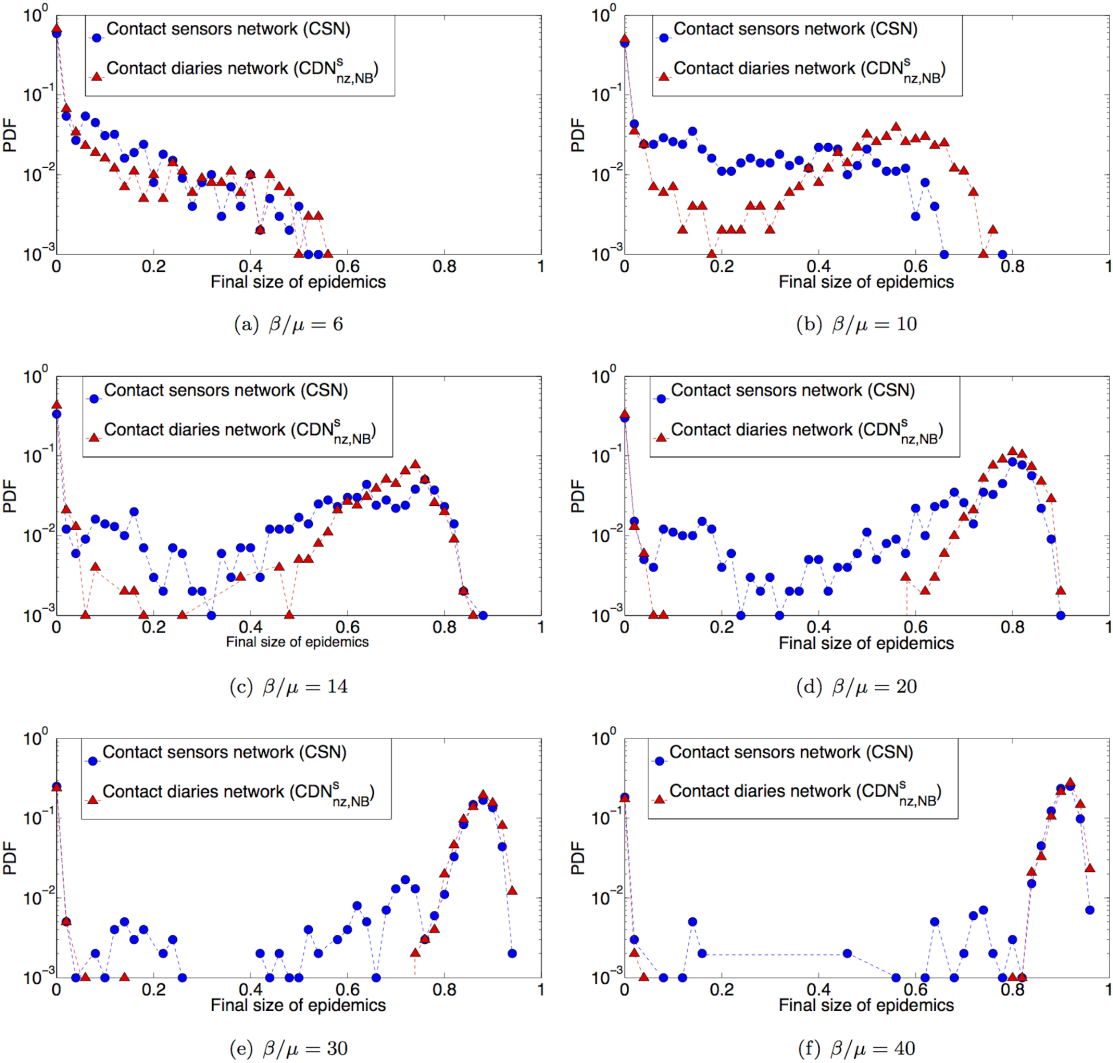
Outcome of the spreading processes. Comparison of the distributions of epidemic sizes obtained for 1000 SIR simulations performed on the contact sensors network and on the surrogate contact network with weights randomly drawn from a negative binomial fit of the distribution of contact durations registered by sensors in several environments (${\text{CDN}}_{\text{nz,NB}}^{s}$). Each process starts with one random infected seed. (a) *β*/*μ* = 6, (b) *β*/*μ* = 10, (c) *β*/*μ* = 14, (d) *β*/*μ* = 20, (e) *β*/*μ* = 30, (f) *β*/*μ* = 40.

For all these datasets, the distributions of cumulated contact durations are broad and, as also discussed in [44], can be modeled by negative binomial functional forms. We show in the Supporting Information that similar parameters of the negative binomial fit are obtained for each dataset and for the combined one. Therefore, to further generalize the procedure and avoid relying on a single dataset, we consider in the following the fit of the five combined datasets. We then assign to the links of the ${\text{CDN}}_{\text{nz}}^{s}$ weights drawn at random from this fitted distribution, obtaining ${\text{CDN}}_{\text{nz,NB}}^{s}$. Figs 7 and 6 compare the distributions of epidemic sizes obtained when the SIR model is simulated on the resulting surrogate network and on the CSN (see also the Supporting Information, in which we moreover show the outcome of simulations for different initial seeds, as well as the temporal evolution of the density of infectious individuals in the population when using the ${\text{CDN}}_{\text{nz,NB}}^{s}$.)

Despite using less information on the specific context than ${\text{CDN}}_{\text{nz,S}}^{s}$, since we do not rely on the specific distribution of weights measured there, the surrogate contact network ${\text{CDN}}_{\text{nz,NB}}^{s}$ leads to a good prediction of the epidemic risk. In particular, the emergence, location and amplitude of the peak of the distribution at large epidemic values is correctly predicted. However, the distribution of final epidemic sizes is systematically shifted towards higher shares of population with respect to the ${\text{CDN}}_{\text{nz,S}}^{s}$ case. Thus, for small values of *β*/*μ* the outcome of simulations using ${\text{CDN}}_{\text{nz,S}}^{s}$ are in slightly better agreement with the CSN case. However, for higher *β*/*μ* and for the largest epidemic size reached, the ${\text{CDN}}_{\text{nz,NB}}^{s}$ performs better. Overall, both surrogate networks yield satisfactory predictions of the epidemic risk associated to a propagation on the CSN.

## Discussion

Data on the contact patterns of individuals collected by different methods lead to different contact network structures, and some studies have started to investigate this issue through detailed quantitative comparisons [21, 36]. In the present paper, we have gone further by comparing the outcome of simulations of spreading processes performed on contact networks gathered either through wearable sensors or through contact diaries. Not surprisingly, we have shown that the results differ strongly, due to the low participation rate to the diaries, the under-reporting of contacts and the overestimation of contact durations in diaries. In particular, the direct use of the links and durations reported in the diaries yields a peculiar distribution of epidemic sizes suggesting a very strong community structure that might lead to the design of inadequate containment strategies. On the other hand, using the links reported in the diaries but more realistic weights yields a strong underestimation of the epidemic risk.

In a second and more important step, we have asked if, despite this low participation rate and these biases, the information contained in the contact network built from the contact diaries can be used to build a surrogate contact network whose properties are close enough to the real contact network (considered here to be the one obtained from the wearable sensors) to yield a correct estimation of the epidemic risk when used in simulations of spreading processes. The rationale comes from the structural similarities found in the contact matrices giving the densities of links between individuals of different classes obtained using both sensors and diaries [21]. These similarities suggest to build a surrogate contact network starting from the contact diary network, adding nodes and links in order to maintain this matrix fixed, and assigning weights to the links. We note that two recent works [37, 40] have considered related but different issues. In [37], only diary data is available, and the authors present a synthetic network model based on data and adjusting for under-reporting. This adjustment for reporting errors leads in this case only to a small difference in epidemic predictions. In [40] on the other hand, only sensor data is considered, and the authors assume to have an incomplete information on the contact network registered by sensors due to an uniform population sampling (hence, all contacts between participating individuals are assumed to be known). Here on the other hand, the available dataset is given by diaries, in which population sampling is not uniform (actually, the students who filled in diaries tend to have more contacts than the others) and in which under-reporting implies that many links between participating individuals are also missing. Moreover, we face two additional issues (i) the low sampling rate yields a contact matrix of link densities which contains zeros, in an unrealistic way, and (ii) various possibilities can be considered when assigning weights to the links of the surrogate networks as weights reported in the diaries are strongly overestimated. Despite these issues, the surrogate network we build yields, when used in simulations, a good agreement with simulations performed on the whole contact sensor network in terms of epidemic risk prediction, under the condition of using the list of weights (cumulative contact durations) gathered by the wearable sensors. In order to get rid of this condition, we argue that this list comes from a distribution that has been shown in previous works to be very robust across contexts [9]. We therefore consider weights taken at random from a pool of publicly available datasets, and show that using these weights gives also satisfactory results. Overall, we thus have presented a procedure that uses only the information contained in the contact diaries and in public data, which allows to obtain a good prediction of the epidemic risk, as measured by the distribution of epidemic sizes, when used in simulations of a spreading process.

In the Supporting Information, we moreover consider the issue of using, instead of contact diaries, data coming from friendship surveys, in order to build the surrogate contact network used in the simulations. We show that the epidemic risk prediction obtained through this procedure is not accurate. This could be expected as daily encounters in the school are not necessarily related to the existence of a relationship between students: contacts occur between non-friends due to daily activities, while friends do not meet necessarily every day. This outcome highlights the importance of taking into account the different nature of social ties [21, 46], which can each be relevant for specific processes.

Some limitations of our work are noteworthy. First, our results rely on an assumption made in replacing the zeros observed in the contact matrix of link densities by random values. In the context under scrutiny, zero values can indeed easily be considered as unrealistic. In other contexts, it might however happen that different groups in the population really do not mix. In such a case, one might expect that this kind of information could be gathered from other sources (schedules, location of classrooms or offices, etc) [47] and thus integrated into the procedure. Second, we have considered as ground truth the contact sensor network. On the one hand, this network in fact suffers from an incomplete participation, so that the outcome of spreading processes is underestimated with respect to hypothetical data containing information on the whole population. However, such underestimation can be compensated through the procedure presented in [40]. On the other hand, it is important to note that it is not yet completely clear whether the contacts measured by wearable sensors are the best proxy for potentially infectious contacts. We work therefore under this hypothesis, which is indeed quite widely used but should be kept in mind. Third, we have considered here static networks. As discussed in [39], the outcome of simulations is then close enough to the one of simulations taking into account the full contact dynamics if we consider slow enough processes. For fast processes, the burstiness of contacts becomes very relevant; in this case, it would be crucial to supplement our procedure by the construction of surrogate timelines of contacts and intervals between contacts at high temporal resolution, as done in [40]. Finally, we cannot at this point investigate the efficiency of our procedure in other contexts, for lack of datasets reporting contacts measured by both wearable sensors and contact diaries in the same context and at the same date. Hopefully such datasets will become more available in the future, yielding new testing grounds for our method. Many populations of interest can indeed be divided into groups or categories that do not mix homogeneously, often with more contacts within groups than between groups, and for which the contact matrix formalism and the procedures we present to construct surrogate networks are therefore relevant [40]. We conclude by mentioning that future work could also investigate other dynamical processes on networks, such as information spreading or opinion formation processes.

## Methods

### Data description

The datasets we use have been presented and made publicly available in [21]. They correspond to contacts between students of 9 classes in a high school in France, collected through wearable sensors on the one hand and contact diaries on the other hand. The sensors registered contacts with a temporal resolution of 20*s* for 327 participating students (out of 379 in the 9 classes, i.e., a 86.3% participation rate) during the week of Dec. 2–6, 2013. Contact diaries contain data reported by students about encounters and their cumulative durations for Dec. 5, 2013. In these diaries, students were asked to report the cumulative durations of their contacts choosing among four intervals: at most 5 minutes, between 5 and 15 minutes, between 15 minutes and 1 hour, more than one hour. The students belong to 9 classes with different specializations: “MP” classes focus more on mathematics and physics, “PC” classes on physics and chemistry, “PSI” classes on engineering studies and “BIO” classes on biology. We collected data among students of nine classes corresponding to the second year of such studies: 3 classes of type “MP” (MP, MP*1, MP*2), two of type “PC” (PC and PC*), one of type “PSI” (PSI*) and 3 of type “BIO” (2BIO1, 2BIO2, 2BIO3).

Using these datasets, we build two networks of contacts among students for the same day (Dec 5, 2013): the Contact Sensors Network (CSN) and the Contact Diaries Network (CDN). In each network, nodes represent students, and a links is drawn between two students if:

1. sensors register at least one contact during the relevant day (CSN);
2. at least one of the two students reported an encounter (CDN).

The resulting networks have 295 nodes for the CSN (other students were absent or did not wear the sensors on that day) and 120 nodes for the CDN. In particular, no student from classes PC* and PSI* filled a diary, and only one from MP*1. We thus discarded these classes in most of the analysis and in particular in the contact matrices, remaining with 6 classes.

Each link carries a weight. In the CSN it represents the cumulative duration of contacts registered by sensors during the day. For the CDN we consider several possibilities. In the CDN_D_ we use weights reported in the diaries: we associate to each time-interval its maximum possible value (5, 15, 60 minutes respectively for the first three intervals and 4 hours for the last one. This choice takes into account data reported and registered and the school schedule) and, if two students reported different durations for their encounter, we use the average of the reported values. In the CDN_S_ on the other hand, we consider weights randomly drawn from the distribution of contact durations registered by sensors. Results are averaged over 1000 such weight assignments. For the CDN_S’_ finally, we start from CDN and rank the *E* links in decreasing order of their reported weights (assigned as in CDN_D_, and with random order for equal weights). We extract *E* weights from the distribution of contact durations registered by sensors, rank them as well in decreasing order, and assign the weights to the links of CDN in such a way to match the two orderings (i.e., assigning the largest weights to the links with largest reported weights).

### Matching networks

The CSN, the CDN_D_ and the CDN_S_ are matched to retain only nodes appearing in both CSN and CDN (see Table 1 for details about classes size before and after matching). We refer to them as the *matched* networks: CSN^*m*^, ${\text{CDN}}_{\text{D}}^{m}$ and ${\text{CDN}}_{\text{S}}^{m}$.

**Table 1:** Comparison of network properties. Number of nodes in each class in the contact sensors network and in the contact diaries network in the original (respectively CSN, CDN) and the matched forms (respectively CSN^*m*^, CDN^*m*^).

	CSN	CDN	CSN^*m*^	CDN^*m*^
**2BIO1**	35	22	20	20
**2BIO2**	30	13	11	11
**2BIO3**	33	15	13	13
**MP**	32	23	23	23
MP*1	28	1	0	0
MP*2	34	19	18	18
**PC**	40	27	23	23
PC*	35	0	0	0
PSI*	28	0	0	0
**Tot**	295	120	108	108

The CMDN is built by using a Contact Matrix Distribution (CMD). Following [44], we consider a CMD where each entry, (*X*, *Y*), is the empirical distribution of durations reported by diaries for contacts between all students in class *X* and class *Y*, including zero durations (corresponding to an absence of link between two students). We fit each such distribution by a negative binomial functional form. Then, for each pair of nodes, we draw at random a weight using the corresponding negative binomial fit. Note that in this way we do not maintain fixed the link structure of the CDN. We however keep on average the same density of links between different classes.

### Construction of surrogate networks

The basic steps for building a binary surrogate contact network CDN^*s*^ for the six considered classes, starting from the matched contact diaries network, are:

1. we add all missing nodes in each class (we know the number of students in each class in the CSN): the number of nodes grows from 108 in the CDN^*m*^ to 204 in the CDN^*s*^;
2. we add new links within and between classes in order to keep fixed the observed contact matrix of edge densities for the contact diaries network (given in Fig 3(b)). To this aim, we randomly pick up pairs of nodes, *i* belonging to class *X* and *j* to *Y*. If *i* and *j* are not yet linked and if the current density of links between classes *X* and *Y* is smaller than the corresponding entry of the empirical matrix (Fig 3(b)), we add a link between *i* and *j*.
3. the previous step is repeated until we obtain link densities within and between classes equal to the ones of the CDN^*m*^ (Fig 3(b)).

Results are averaged over 500 realisations of this procedure.

As explained in the main text, we moreover deal in two different ways with the zero values of the link densities between several class-pairs in the CDN. We either keep these densities or replace them with values drawn at random from a uniform distribution of values between the minimum and maximum values (diagonal excluded) of the contact matrix of Fig 3(b). In this way the contact matrix structure is preserved, with more interactions within than between classes.

To assign weights to the links of the surrogate networks, we consider several possibilities. We first assume homogeneous contact durations and assign to each link a weight equal to the average of cumulative durations registered by sensors. This yields two versions of the surrogate contact networks:

- ${\text{CDN}}_{\text{z,H}}^{s}$: with homogeneous contact durations and keeping zero densities in the contact matrix of edge densities;
- ${\text{CDN}}_{\text{nz,H}}^{s}$: with homogeneous contact durations and zero densities replaced in the contact matrix of edge densities.

We refer to the contact sensors network under the homogeneous duration hypothesis by CSN_H_.

If instead we assume heterogeneous contact durations, we obtain two possible surrogate contact diaries networks: we assign weights at random to the links of CDN^*s*^, drawn at random with replacement from the list of durations either reported by students or registered by sensors. We thus obtain four versions of the surrogate contact networks:

- ${\text{CDN}}_{\text{z,D}}^{s}$, with durations drawn from the ones reported by students and keeping zero densities in the contact matrix of edge densities;
- ${\text{CDN}}_{\text{nz,D}}^{s}$, with durations drawn from the ones reported by students and zero densities replaced in the contact matrix of edge densities;
- ${\text{CDN}}_{\text{z,S}}^{s}$, with durations drawn from the ones registered by sensors and keeping zero densities in the contact matrix of edge densities;
- ${\text{CDN}}_{\text{nz,S}}^{s}$, with durations drawn from the ones registered by sensors and zero densities replaced in the contact matrix of edge densities.

Finally, the surrogate contact network obtained by assigning weights randomly drawn from the negative binomial fit of the distribution of publicly available contact durations registered by sensors is indicated by the acronym ${\text{CDN}}_{\text{nz,NB}}^{s}$.

## Supporting Information

S1 File Supplementary pdf file containing all supplementary figures and tables. (PDF)
