# Supplementary material for: How to Estimate Epidemic Risk from Incomplete Contact Diaries Data?
Source: PLoS Comput Biol. 2016 Jun 24;12(6):e1005002. doi: 10.1371/journal.pcbi.1005002 (PMC4920368; doi:10.1371/journal.pcbi.1005002)
Supplement: S1 File — (PDF) [file pcbi.1005002.s001.pdf]

How to estimate epidemic risk from incomplete contact diaries data? Supplementary Information

Rossana Mastrandrea[1,2], Alain Barrat[1,3*]
[1] *Aix Marseille Université, Université de Toulon, CNRS, CPT, UMR 7332, 13288 Marseille, France*
[2] *IMT Institute of Advanced Studies, Lucca, Italy*
[3] *Data Science Laboratory, ISI Foundation, Torino, Italy*
∗ *E-mail: alain.barrat@cpt.univ-mrs.fr*

1 Structure of the networks and of the contact matrices

1.1 Networks' main characteristics

Tables S1-S2 and Fig. S1 report some standard statistical characteristics of the empirical and surrogate networks considered in the main text. Moreover, in Tab. S2 and Fig. S1, we consider the versions of CSN and CDN restricted to 6 classes, and we use unweighted measures, therefore we consider the surrogate networks $\mathrm{CDN}^s_{\mathrm{z}}$ and $\mathrm{CDN}^s_{\mathrm{nz}}$ without referring to a weight assignment.

The contact diaries network has less nodes and links than the contact sensor network. Moreover, the average degree is much smaller, with the whole degree distribution shifted to smaller values. The maximal cliques are also smaller, while the shortest paths are longer in the CDN. All this is consistent with its more dilute structure with in particular less connections between classes. This also leads to more extreme values of the betweenness centrality (corresponding to the bridges between classes).

The $\mathrm{CDN}^s_{\mathrm{z}}$ surrogate network becomes closer to the contact sensor network for the degree distribution and the shortest path lengths. As the zero values in the contact matrix are not replaced in this case, it however keeps a very strong modular structure, with large values of the betweenness centrality and larger shortest path lengths than the CSN. The $\mathrm{CDN}^s_{\mathrm{nz}}$ on the other hand yields shortest path lengths in perfect agreement with the CSN structure and closer distributions of degrees and betweenness centralities (properties of crucial importance in terms of propagation processes. As the construction of the $\mathrm{CDN}^s_{\mathrm{nz}}$ does not take into account clustering or cliques, these properties remain however smaller than for the original CSN.

	**CSN**	**CDN**
**Nodes**	295	120
**Links**	2162	348
**Density**	0.05	0.05
**Avg. Degree**	15(8)	6(2)
**Avg. Clustering**	0.38	0.45
**Avg. Betweenness**	532(797)	519(983)
**Avg. SPL**	2.81(0.8)	5.36(2.73)
**Maximal Clique Size**	9	5

Tab. S1: **Comparison of some properties of the initial contact sensor network and surrogate contact diaries network.** SPL: shortest path length. In parenthesis, standard deviations.

	**CSN** (6 classes)	**CDN** (6 classes)	$\mathbf{CDN}^s_\mathbf{z}$	$\mathbf{CDN}^s_\mathbf{nz}$
**Nodes**	204	119	204	204
**Links**	1600	347	1076	1324
**Density**	0.08	0.05	0.05	0.06
**Avg. Degree**	16(7)	6(2)	10(4)	12(4)
**Avg. Clustering**	0.44	0.45	0.25	0.2
**Avg. Betweenness**	328(523)	515(974)	544(1024)	333(264)
**Avg. SPL**	2.61(0.8)	5.37(2.74)	3.7(1.6)	2.64(0.74)
**Maximal Clique Size**	9	5	6	6

Tab. S2: **Comparison of some properties of the contact sensor network and contact diaries network reduced to 6 classes, and for the surrogate contact diaries networks obtained either keeping the zero elements in the contact matrix, or replacing them.** SPL: shortest path length. In parenthesis, standard deviations.

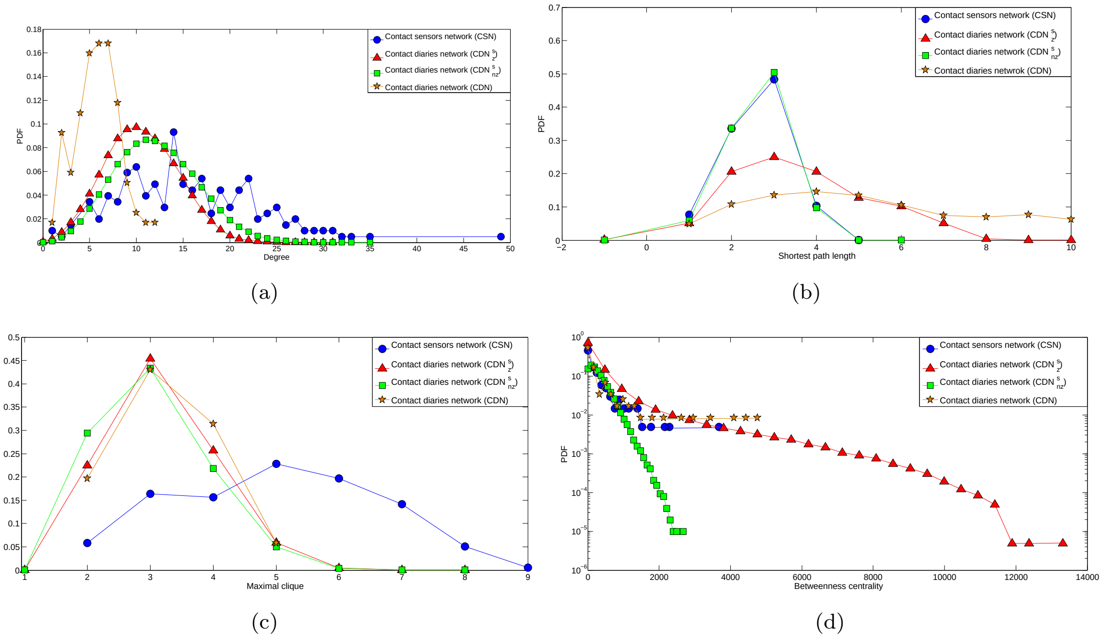

Fig. S1: **Networks statistical properties.** Distributions of degrees, shortest path lengths, betweenness centralities and maximal clique sizes for the contact sensor network and the contact diaries network reduced to 6 classes, as well as for the surrogate networks.

1.2 Original contact sensors and diaries networks

In Fig. S2 we report the contact matrices of average durations of contacts between students of different classes. Each entry of the matrix, C_{XY}, is given by the average of the total contact durations between students in class X and students in class Y.

The contact sensors network and the contact diaries network with weights reported by students have a diagonal structure: people in the same class are in general more likely to stay in contact longer than people belonging to different classes (except for MP*1 for which only one student filled in the diaries). In the $\mathrm{CDN_S}$ case, this structure is not respected due to the random assignment of weights drawn from the distribution of durations registered by sensors (Fig. S2 (c), (d), (e)).

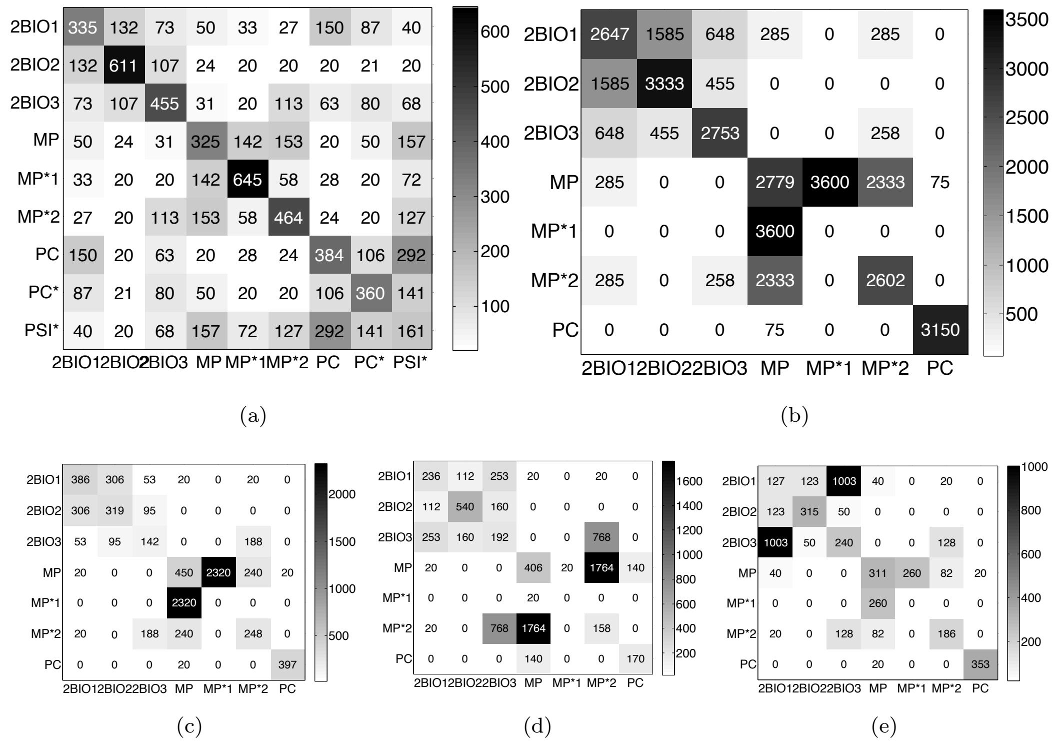

Fig. S2: **Contact matrices of average durations.** (a) Contact sensors network (CSN); (b) Contact diaries network with durations reported by students ($\mathrm{CDN_D}$); (c), (d), and (e) three examples of contact diaries network with weights randomly drawn from the distribution of contact durations registered by sensors ($\mathrm{CDN_S}$).

1.3 Matched contact sensors and diaries networks

Here, we report the contact matrices of average durations of contacts for the matched versions of the contact sensors and diaries networks. The weighted diagonal structure is evident in the CSN^m, $\mathrm{CDN}^m_{\mathrm{D}}$ and CMDN (respectively Fig.s S3 (a), (b) and (d)), while it is not respected in the $\mathrm{CDN}^m_{\mathrm{S}}$ case. This depends again on the random assignment of durations registered from sensors to the links reported in the diaries.

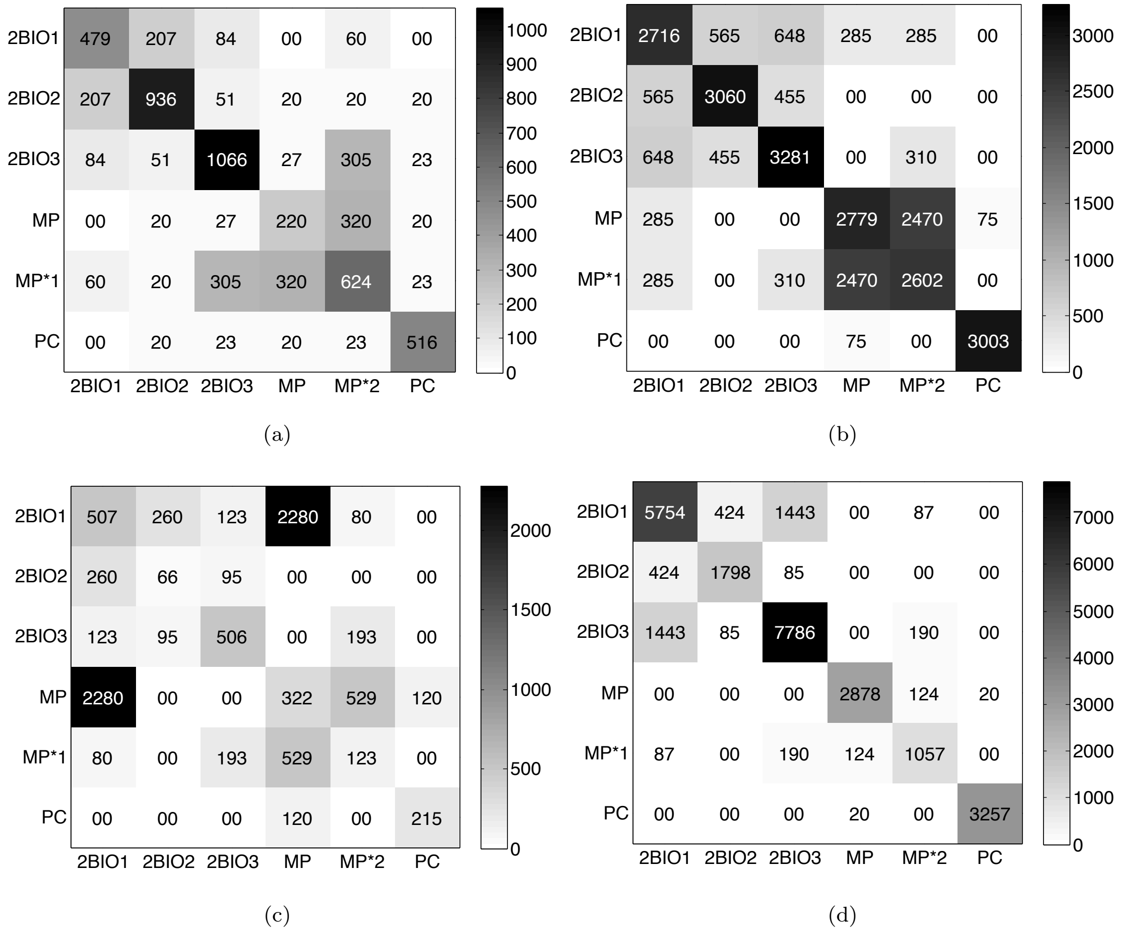

Fig. S3: **Contact matrices of average durations for matched networks.** (a) Contact sensors network CSN^m; (b) Contact diaries network with durations reported by students ($\mathrm{CDN}^m_{\mathrm{D}}$); (c) one example of contact diaries network with weights randomly drawn from the distribution of contact durations registered by sensors ($\mathrm{CDN}^m_{\mathrm{S}}$); (d) one example of contact diaries network with weights (including zero values, corresponding to an absence of link), randomly drawn from the negative binomial fit of the distributions of durations reported by students within and between classes (CMDN).

2 Impact of the initial seed

We show in Fig.s S4, S5 and S6 how the properties of the spread obtained in the various considered networks depend on the class of the initial seed: the distribution of the number of secondary infections per index case, the temporal evolution of the density of infectious individuals, and the distribution of the final size of the epidemics.

Interestingly, the peculiar structure of the contact diaries network leads to a non-trivial dependence on the initial seed. In particular, if the seed is in PC, which is little connected to the other classes, the number of secondary infections is larger (Fig. S4), leading to an initially faster spread (S5) that however decreases also much faster (as with high probability it does not manage to reach the other classes). Some less marked dependency from the initial seed is observed for the CSN, with a slower spread if the initial seed is in MP. The surrogate network also yields a slower spread in this case, even if the effect is not as strong, and the peculiar shape of the incidence for an initial seed in PC is not observed.

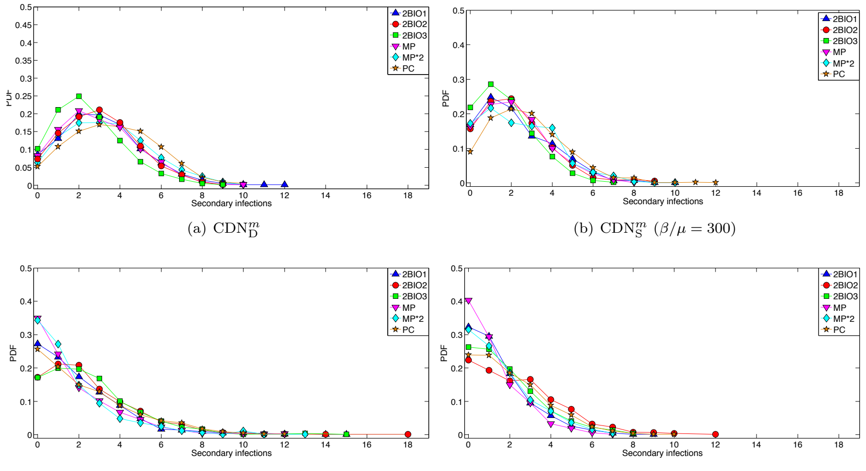

(a) $\mathrm{CDN}^m_{\mathrm{D}}$ (b) $\mathrm{CDN}^m_{\mathrm{S}}$ ($\beta/\mu = 300$)

(c) CSN (d) $\mathrm{CDN}^s_{\mathrm{nz,NB}}$

Fig. S4: **Distribution of the number of secondary infections by the initial seed.** Distributions are averaged over 1000 realizations and separated depending on the class of the initial seed, for the contact diaries networks with weights durations respectively reported by students ($\mathrm{CDN}^m_{\mathrm{D}}$) and registered by sensors $\mathrm{CDN}^m_{\mathrm{S}}$), the contact sensor network restricted to 6 classes and the surrogate network $\mathrm{CDN}^m_{\mathrm{nz,NB}}$. $\beta/\mu = 30$.

With respect to the overall impact of the spread, as measured by the distributions of final sizes of epidemics, the situation is more striking. For the contact diaries network, the case of a seed in PC is once again singular, reaching only small sizes with large probability (as the spread is "trapped" in PC and does not easily reach other classes). For the $\mathrm{CDN}^m_{\mathrm{D}}$ and $\mathrm{CDN}^m_{\mathrm{S}}$ we also observe similar distributions for seeds in one of the three biology classes, which are indeed well connected, and a slightly different distribution when the seed is in one of the MP classes. For CSN and the surrogate network on the other hand, no clear dependency on the initial seed is observed for the distributions of the final size of epidemics: the differences observed for the contact diaries network are thus spurious, and the use of the surrogate networks corrects this spurious effect.

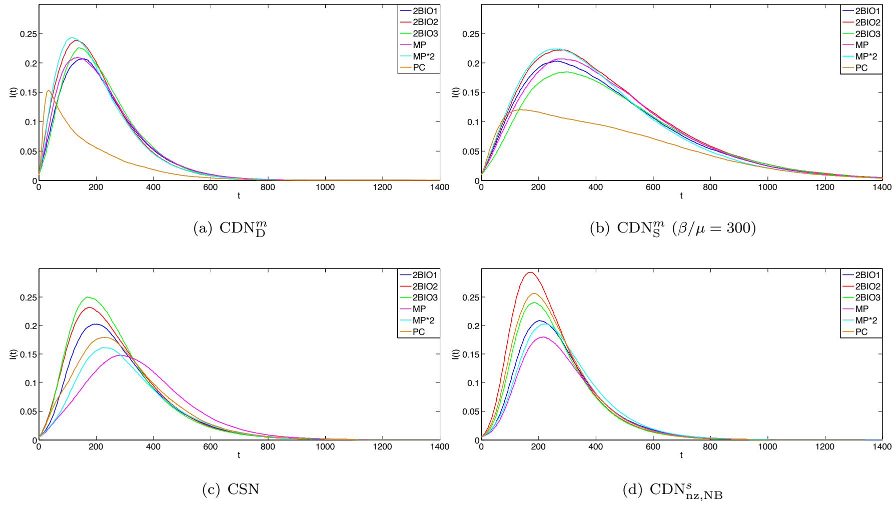

(a) $\mathrm{CDN}^m_{\mathrm{D}}$ (b) $\mathrm{CDN}^m_{\mathrm{S}}$ ($\beta/\mu = 300$)

(c) CSN (d) $\mathrm{CDN}^s_{\mathrm{nz,NB}}$

Fig. S5: **Temporal evolution of the fraction of infectious individuals.** We show the evolution with time of the density of infectious individuals, averaged over 1000 realizations and separated depending on the class of the initial seed, for the contact diaries networks with weights durations respectively reported by students ($\mathrm{CDN}^m_{\mathrm{D}}$) and registered by sensors $\mathrm{CDN}^m_{\mathrm{S}}$), the contact sensor network restricted to 6 classes and the surrogate network $\mathrm{CDN}^m_{\mathrm{nz,NB}}$. In order to show the effect of the seed for the $\mathrm{CDN}^m_{\mathrm{S}}$, we use a large value of $\beta/\mu = 300$, while for the other cases $\beta/\mu = 30$.

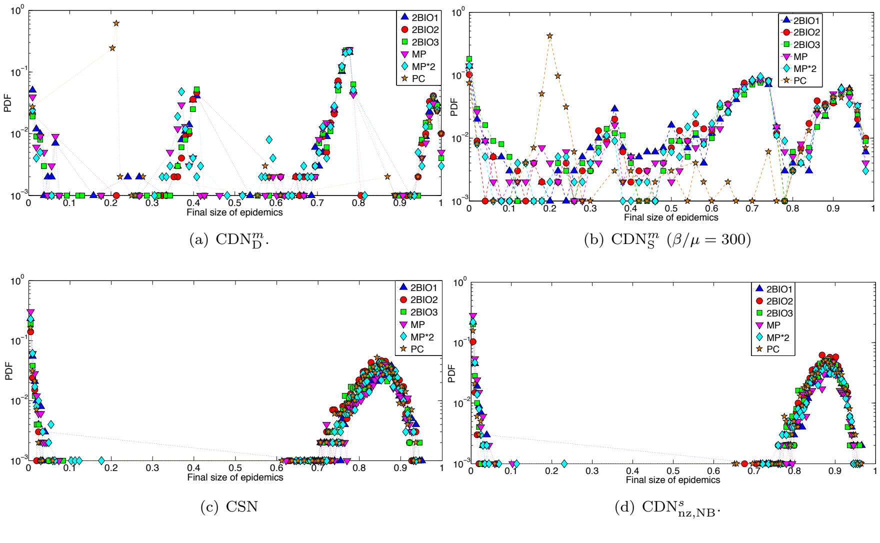

(a) $\mathrm{CDN}^m_{\mathrm{D}}$. (b) $\mathrm{CDN}^m_{\mathrm{S}}$ ($\beta/\mu = 300$)

(c) CSN (d) $\mathrm{CDN}^s_{\mathrm{nz,NB}}$.

Fig. S6: **Impact of the initial seed.** Distribution of final sizes of final sizes of epidemics, separated depending on the class of the initial seed, for the contact diaries networks with weights durations respectively reported by students ($\mathrm{CDN}^m_{\mathrm{D}}$) and registered by sensors $\mathrm{CDN}^m_{\mathrm{S}}$), the contact sensor network restricted to 6 classes and the surrogate network $\mathrm{CDN}^m_{\mathrm{nz,NB}}$. In order to show the effect of the seed for the $\mathrm{CDN}^m_{\mathrm{S}}$, we use a large value of $\beta/\mu = 300$, while for the other cases $\beta/\mu = 30$.

3 Spreading simulations using the CMDN

In figure S7 we show the distribution of final epidemic sizes for SIR simulations performed on the CSN^m and the CMDN. The two networks have the same nodes but different links and weights. Weights in the CMDN are randomly drawn from distributions obtained from a negative binomial fit, within and between classes, performed on the distributions of durations reported by students. We chose several values of parameters β and μ to check the robustness of the outcome shown in Fig. 2 in the paper. In Fig. S8 we compare the boxplots of the two distributions for epidemic sizes larger than 10%.

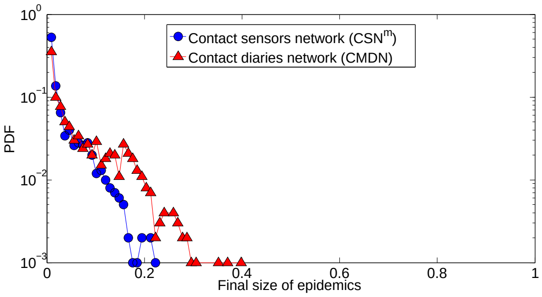

(a) $\beta/\mu = 6$

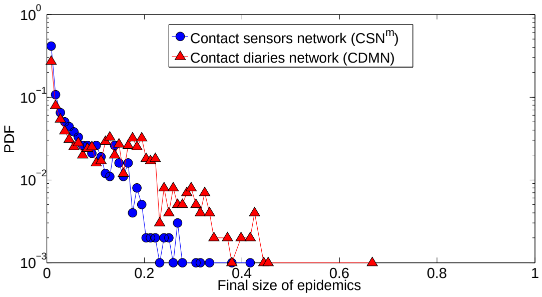

(b) $\beta/\mu = 10$

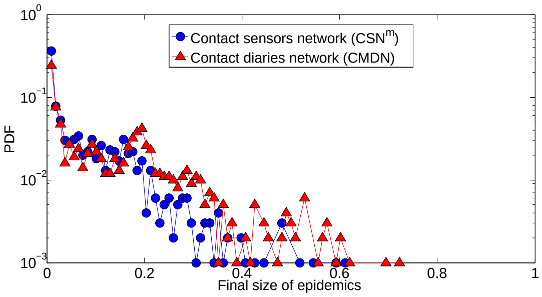

(c) $\beta/\mu = 14$

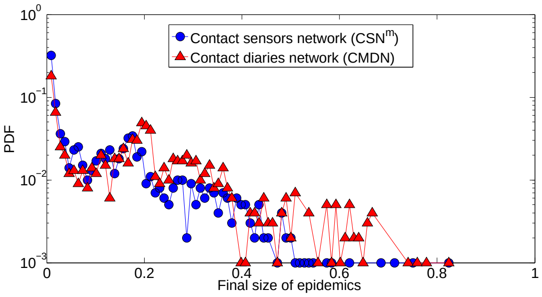

(d) $\beta/\mu = 20$

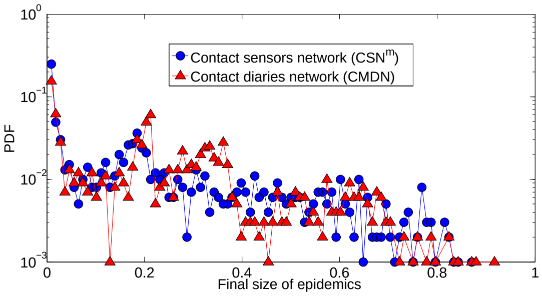

(e) $\beta/\mu = 30$

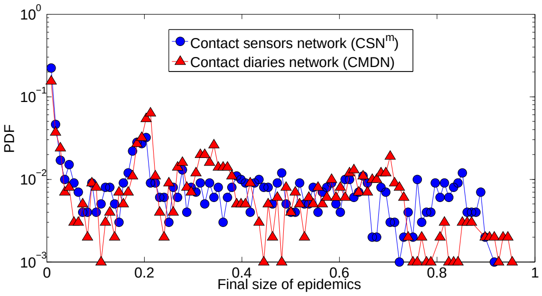

(f) $\beta/\mu = 40$

Fig. S7: **Distributions of final size of epidemics.** Outcome of 1000 SIR simulations performed on the contact sensors network (CSN^m) and the CMDN obtained with weights randomly drawn from the negative binomial fit of distributions of contact durations within and between classes registered by sensors. Each process starts with one random infected seed.

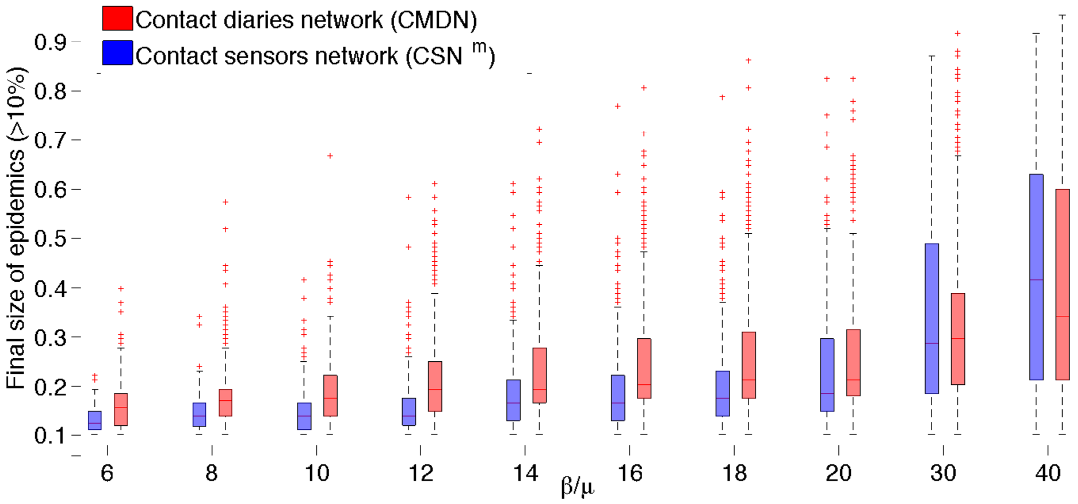

Fig. S8: **Box-plot of final size of epidemics.** Comparison of the distributions of final size of epidemics, for sizes larger than 10%, for SIR simulations performed on the contact sensors network (CSN^m) and the CMDN obtained with weights randomly drawn from the negative binomial fit of the distribution of contact durations within and between classes registered by sensors. For each box, the central mark stands for the median, its edges represent the 25th and 75th percentiles. The whiskers extend to the most extreme data points not considered outliers, while outliers are plotted individually (1000 simulations for each value of β/μ).

4 Original, reduced and reshuffled contact sensors networks

In Fig. S9 we explore the change in the prediction of the epidemic risk associated to the contact sensors network when some classes are removed from the sample. This choice depends on the fact that such classes are absent or under-represented by the contact diaries network, and so for them a construction of surrogate links using the available information would be impossible. The original network has 295 nodes and 2162 links, its reduced form has 204 nodes and 1600 links.

The shape of the distributions are quite similar with an overestimation of the epidemic peak for the reduced network with respect to the original contact sensors network. This outcome suggests that the removed classes are not well connected with the rest of the population, preventing the diffusion of the epidemics and reducing the epidemic risk.

In Fig. S10 we show the outcome of SIR simulations on the reduced contact sensors network and on a null-model with the same link structure but reshuffled weights. The comparison between the two distributions reveals a good agreement for the peak of the distribution, while the probability to observe epidemics involving intermediate shares of population is almost zero when weights are reshuffled. This suggests that the occurrence of epidemics of intermediate size depends on specific correlations between structure and weights.

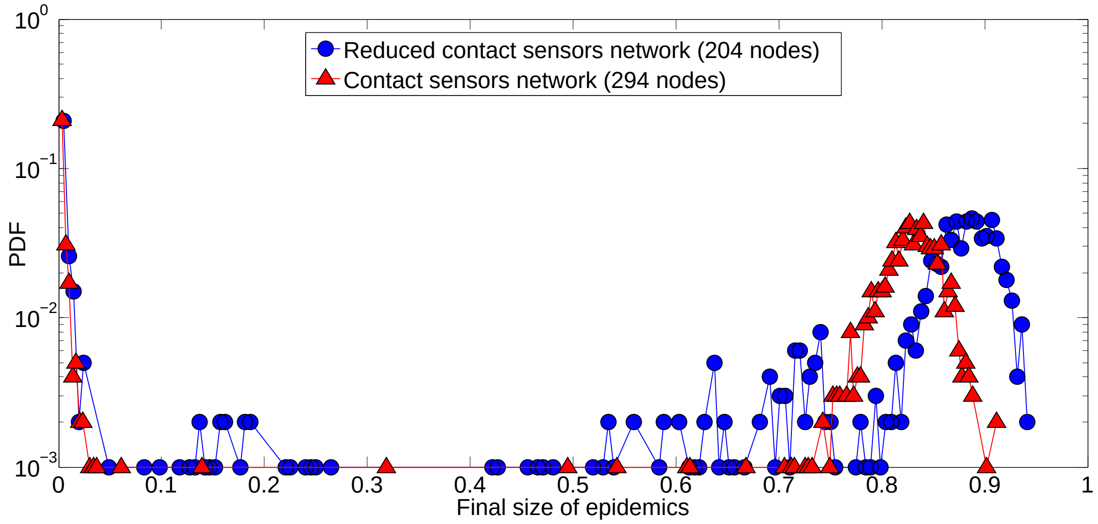

Fig. S9: **Distribution of final size of epidemics.** Comparison between the distributions obtained from 1000 SIR simulations performed on the original contact sensors network and its reduced form obtained by removing the three classes not or under-represented in the contact diaries network. Each process starts with one random infected seed. $\beta/\mu = 30$.

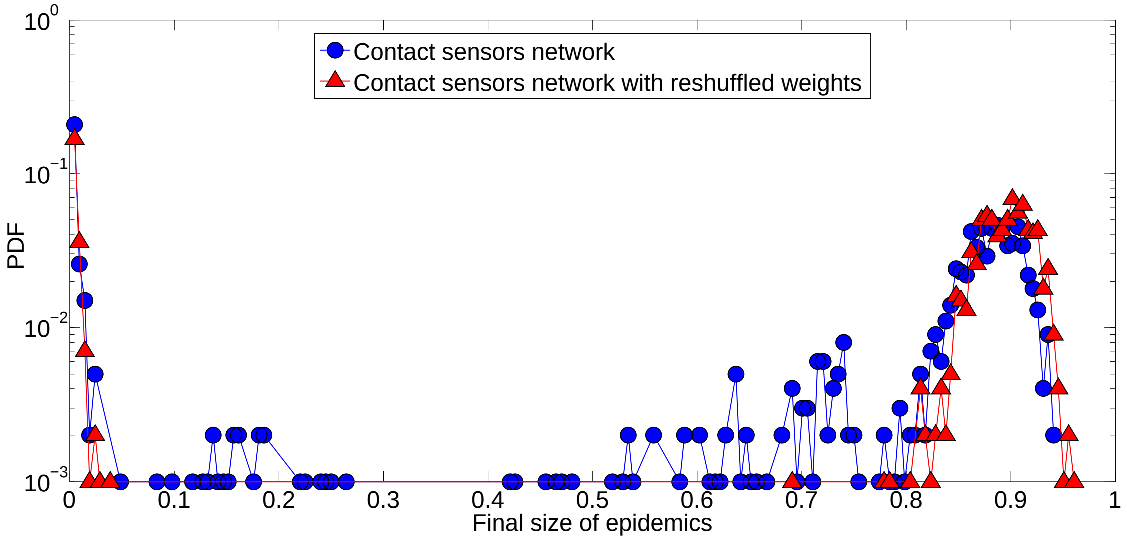

Fig. S10: **Distribution of final size of epidemics.** Comparison of the distributions obtained from 1000 SIR simulations performed on the reduced contact sensors network and on a null model with the same binary structure but reshuffled weights. Each process starts with one random infected seed. $\beta/\mu = 30$.

5 Simulations on surrogate networks

5.1 Surrogate contact network with homogeneous durations

Figure S11 complements the result presented in Fig. 4 of the main text on the comparison between the contact sensors network and the surrogate contact network under the hypothesis of homogeneous cumulative durations. Here, we focus on the share of epidemic processes reaching (a) more than 10%, (b) between 20% and 70%.

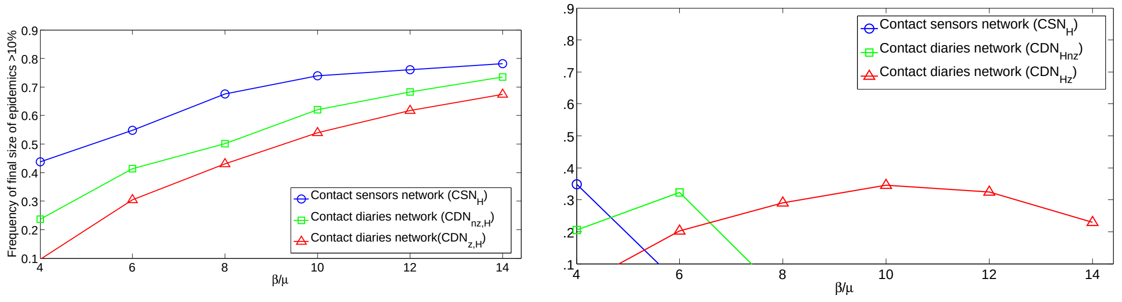

(a) (b)

Fig. S11: **Fraction of epidemics reaching specific shares of the population.** Frequency of processes involving (a) more than 10% and (b) between 20% and 70% of the whole population for the contact sensors network ($\mathrm{CSN_H}$) and the surrogate contact networks under the hypothesis of homogeneous cumulative durations ($\mathrm{CDN}^s_{\mathrm{z,H}}$ and $\mathrm{CDN}^s_{\mathrm{nz,H}}$) . Values computed over 1000 SIR simulations. Each process starts with one random infected seed.

5.2 Surrogate contact diaries network with weights registered by sensors

Figure S12 complements the result presented in Fig. 6 of the main text on the comparison between the contact sensors network and the surrogate contact network. As in Fig. S11, we focus on the share of epidemic processes reaching (a) more than 10%, (b) between 20% and 70%.

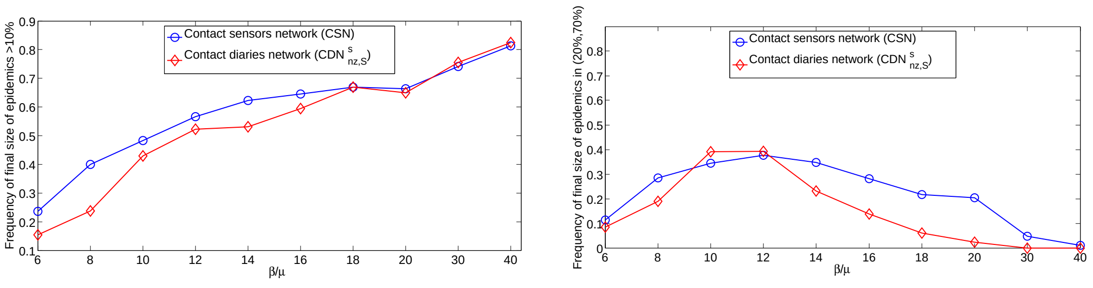

(a) (b)

Fig. S12: **Fraction of epidemics reaching specific shares of the population.** Fraction of simulations involving (a) more than 10% and (b) between 20% and 70% of the whole population for the contact sensors network and the surrogate contact network ($\mathrm{CDN}^s_{\mathrm{nz,S}}$). Values computed over 1000 SIR simulations. Each process starts with one random infected seed.

5.3 Surrogate contact network with durations taken from publicly available data

Table S3 reports the parameters (and related confidence intervals) of a negative binomial fit performed on the distribution of (non-zero) durations registered by sensors for several datasets. There

are several equivalent ways to describe the functional form of a negative binomial distribution, here we use the following definition for a random variable x and parameters r and p. In general, when r is an integer:

$$y = f(x|r,p) = \binom{r+x-1}{x} p^r q^x I_{\{0,1,\dots\}}(x)$$

If r is not integer, the binomial coefficient is replaced by the equivalent expression (using the gamma function):

$$\frac{\Gamma(r+x)}{\Gamma(r)\Gamma(x+1)}$$

Dataset	Parameters
**High-school** [1]	$(0.0137, 0.0002)$<br>([0.001329,0.001404], [0.00018, 0.00021])
**American primary school** [2]	$(0.0455, 0.0005)$<br>([0.04470,0.04621],[0.00044,0.00047])
**French primary school** [3]	$(0.0483, 0.0005)$<br>([0.04710,0.04943],[0.00049,0.00058])
**Office building** [4]	$(0.0276, 0.0006)$<br>([0.02543, 0.02970],[0.00044,0.00066])
**Conference** [5]	$(0.0187, 0.0011)$<br>([0.01828,0.01909],[0.00102,0.00113])
**Hospital** [6]	$(0.2036, 0.0001)$<br>([0.1704,0.2368],[0.00007,0.00014])
**Combined datasets**	(0.0265,0.0004)<br>([0.02622,0.02678],[0.00040,0.00044])

Tab. S3: **Parameters of the negative binomial fits.** For each dataset, we fitted the distribution of contact durations by using a maximum likelihood estimates (MLEs) for parameters r and p. In the first row we report the parameters describing the negative binomial distribution, r and p respectively, while in the second row we show the related 95% confidence intervals.

Fig. S13 complements the results of Fig. 7 of the main text concerning the distributions of epidemic sizes for SIR simulations performed on the contact sensors network and the surrogate contact network with weights randomly drawn from the negative binomial fit of the publicly available distributions of contact durations registered by sensors, for several values of β/μ.

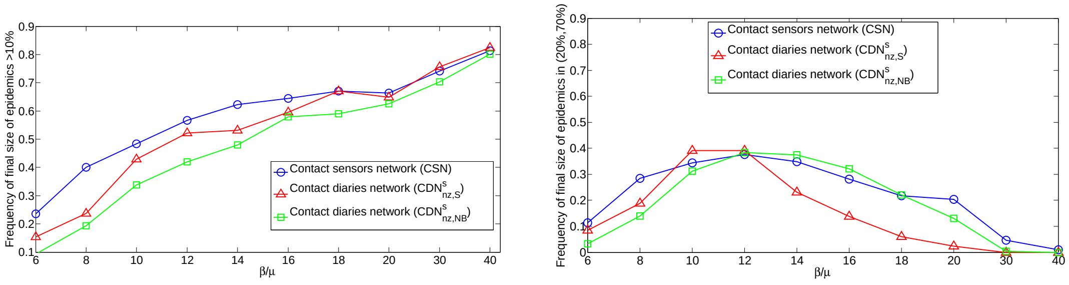

(a) (b)

Fig. S13: **Fraction of epidemics reaching specific shares of the population.** Fraction of epidemics involving (a) more than 10% and (b) between 20% and 70% of the whole population for the contact sensors network and the surrogate contact network ($\mathrm{CDN}^s_{\mathrm{nz,NB}}$). Values computed over 1000 SIR simulations. Each process starts with one random infected seed.

6 Case of the friendship survey

We apply here the same procedure to another survey collected in the same school about friendship among students. Since friendship can be considered time-invariant in a short period such as a week, we focus on two contact sensors networks: a daily network as in the main text and a weekly aggregated network.

Indeed, a high cosine similarity is obtained between the contact matrices of edge densities for the contact and the friendship networks for both cases (respectively 89% and 95%). Here we show that such reported relations are poorly informative in terms of the estimation of epidemic risk in the considered context, with respect to contact diaries. This can be related to the fact that they capture a different type of social interaction not necessarily connected to the number or duration of contacts in the school [1]. This result is in agreement with a recent paper [7] showing that friendship networks are not able to predict individual risk. Note however that [7] focuses on identifying at-risk individuals while we consider here the global risk of the population, as quantified by the distribution of epidemic sizes.

In Fig. S14 and S15 indeed, we compare the distributions of final size of epidemics for SIR simulations performed on the contact sensors network and on surrogate networks obtained through the same procedure as in the main text but taking as starting point the friendship network ($FCN^{s}_{nz,S}$).

The distributions of final size of epidemics for the daily surrogate friendship network shows a clear overestimation of the epidemic risk, with a narrow peak concentrated around high shares of population. On the contrary, if the weekly friendship network is used to build the surrogate network (Figs. S16 and S17) we find an important underestimation of the epidemic risk and a distribution mostly concentrated around small shares of population. Despite the high cosine similarity between the contact matrices of networks, the two social ties (friendship and physical proximity) imply a different underlying structure of interactions which does not allow to have a good prediction of the epidemic risk associated to the network of contacts between students.

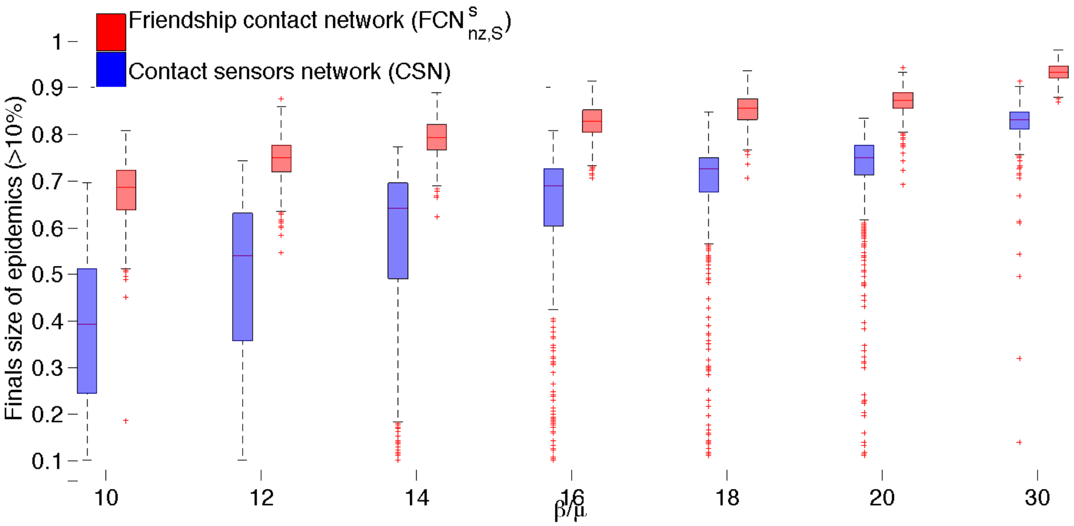

Fig. S14: **Box-plots of the distribution of final epidemic sizes.** Comparison of the distributions obtained from 1000 SIR simulations performed on the contact sensors network (CSN) and the surrogate friendship networks without zero-densities in the contact matrix of link densities and with weights randomly drawn from the distribution of contact durations registered by sensors ($FCN^{s}_{nz,S}$). For each box, the central mark stands for the median, its edges represent the 25th and 75th percentiles. The whiskers extend to the most extreme data points not considered outliers, while outliers are shown individually.

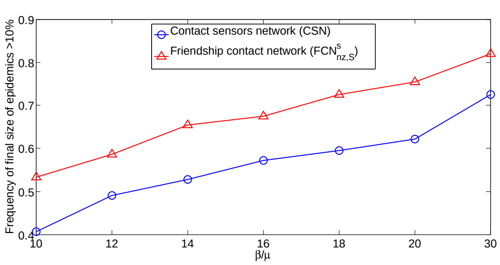

(a)

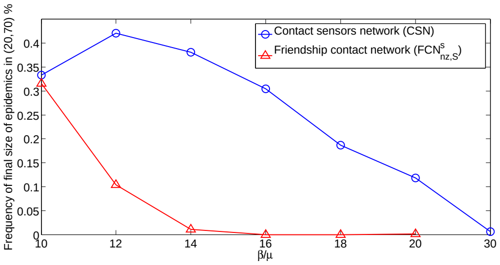

(b)

Fig. S15: **Fraction of epidemics reaching specific shares of the population.** Fraction of epidemics involving (a) more than 10% and (b) between 20% and 70% of the whole population for the contact sensors network (CSN) and the surrogate friendship network ($FCN^{s}_{nz,S}$). Values computed over 1000 SIR simulations. Each process starts with one random infected seed.

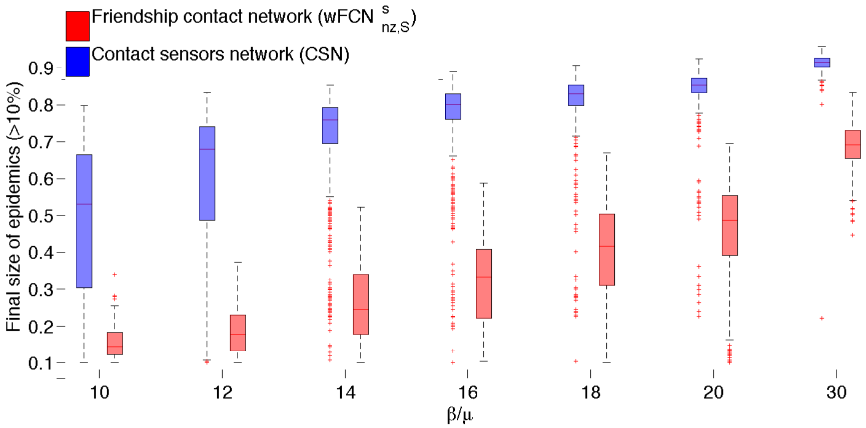

Fig. S16: **Box-plots of the distribution of final epidemic sizes.** Comparison of the distributions of final sizes of epidemics for 1000 SIR simulations performed on the weekly contact sensors network (wCSN) and the weekly surrogate friendship networks without zero-densities and with weights randomly drawn from the distribution of contact durations registered by sensors ($\mathrm{wFCN}^{s}_{\mathrm{nz,S}}$). In each box, the central mark stands for the median, its edges represent the 25th and 75th percentiles. The whiskers extend to the most extreme data points not considered outliers, while outliers are plotted individually.

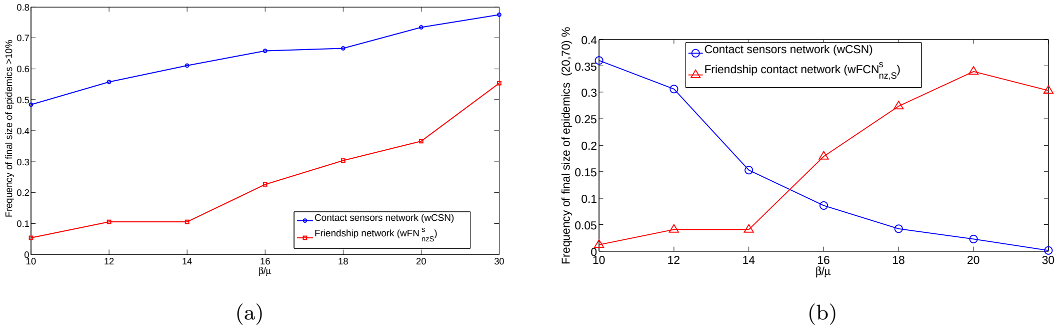

Fig. S17: **Fraction of epidemics reaching specific shares of the population.** Fraction of epidemics involving (a) more than 10% and (b) between 20% and 70% of the whole population for the weekly contact sensors network (wCSN) and the weekly surrogate friendship network ($\mathrm{wFCN}^{s}_{\mathrm{nz,S}}$). Values computed over 1000 SIR simulations. Each process starts with one random infected seed.

References

[1] Mastrandrea,R., Fournet, J., Barrat, A. (2015) Contact patterns in a high school: a comparison between data collected using wearable sensors, contact diaries and friendship surveys. PLoS ONE, 10(9):e0136497.

[2] Toth, D.J.A., Leecaster, M., Pettey, W.B.P., Gundlapalli, A.V., Gao, H., Rainey, J,J., Uzicanin, A., Samore, M.H. (2015). The role of heterogeneity in contact timing and duration in network models of influenza spread in schools. Journal of The Royal Society Interface, 12(108), 20150279 (2015).

[3] Stehlé J., Voirin, N., Barrat, A., Cattuto, C., Isella, L., Pinton, J. F., Quaggiotto, M., Van den Broeck, W., Régis, C., Lina, B., Vanhems, P. (2011). High-resolution measurements of face-to-face contact patterns in a primary school. PLoS ONE 6(8):e23176.

[4] Génois, M., Vestergaard, C., Fournet, J., Panisson, A., Bonmarin, I., Barrat ,A. (2015) Data on face-to-face contacts in an office building suggest a low-cost vaccination strategy based on community linkers. Network Science 3:326.

[5] Stehlé, J., Voirin, N., Barrat, A., Cattuto, C., Colizza, V., Isella, L., Régis C., Pinton, J.F., Khanafer, N., Van den Broeck, W., Vanhems, P. (2011). Simulation of an SEIR infectious disease model on the dynamic contact network of conference attendees. BMC medicine, 9(1), 87.

[6] Mastrandrea, R., Soto-Aladro, A., Brouqui, P., Barrat, A. (2015) Enhancing the evaluation of pathogen transmission risk in a hospital by merging hand-hygiene compliance and contact data: a proof-of-concept study. BMC Research Notes 8:426.

[7] Coviello, L., Franceschetti, M., Rahwan, I. (2015) Limits of Friendship Networks in Predicting Epidemic Risk. arXiv:1509.08368.
